# CRISPR-SKIP: programmable gene splicing with single base editors

**DOI:** 10.1186/s13059-018-1482-5

**Published:** 2018-08-15

**Authors:** Michael Gapinske, Alan Luu, Jackson Winter, Wendy S. Woods, Kurt A. Kostan, Nikhil Shiva, Jun S. Song, Pablo Perez-Pinera

**Affiliations:** 1 Department of Bioengineering, University of Illinois at Urbana-Champaign, 1406 West Green Street, Urbana, 61801-2918 IL USA; 2 Department of Physics, University of Illinois at Urbana-Champaign, 1110 West Green Street, Urbana, IL 61801-3080 USA; 3 Carl R. Woese Institute for Genomic Biology, University of Illinois at Urbana-Champaign, Urbana, IL 61801 USA; 4 Carle Illinois College of Medicine, Champaign, IL 61820 USA

**Keywords:** Gene editing, Base editing, Exon skipping, Alternative splicing, Synthetic biology, CRISPR-Cas9, *RELA*, *PIK3CA*, *BRCA2*, Gene isoform

## Abstract

CRISPR gene editing has revolutionized biomedicine and biotechnology by providing a simple means to engineer genes through targeted double-strand breaks in the genomic DNA of living cells. However, given the stochasticity of cellular DNA repair mechanisms and the potential for off-target mutations, technologies capable of introducing targeted changes with increased precision, such as single-base editors, are preferred. We present a versatile method termed CRISPR-SKIP that utilizes cytidine deaminase single-base editors to program exon skipping by mutating target DNA bases within splice acceptor sites. Given its simplicity and precision, CRISPR-SKIP will be broadly applicable in gene therapy and synthetic biology.

**Electronic supplementary material:**

The online version of this article (10.1186/s13059-018-1482-5) contains supplementary material, which is available to authorized users.

## Background

Programmable nucleases have been used to introduce targeted modifications within a native genomic DNA context [1]. While multiple nuclease architectures have been successfully utilized for genome editing, the clustered regularly interspaced short palindromic repeats (CRISPR)-associated (Cas) system [2–4] has rapidly become the most popular approach because of its flexibility, versatility, and efficacy. CRISPR-Cas9 gene editing is typically accomplished by introducing double-strand breaks (DSBs) at target sites in genomic DNA, which are most commonly repaired by non-homologous end-joining (NHEJ), a mutagenic pathway that creates random insertions and deletions that can be used to knockout genes [1]. However, concerns over off-target mutations and stochastic outcomes of NHEJ-based editing methods [5] have elicited the development of Cas9 isoforms that introduce DSBs with improved specificity [6–8] or even novel technologies that do not rely on the stochastic repair of DSBs, such as single-base editors that can generate C>T or A>G conversions [9–14]. Given their precision and enhanced control over the gene-editing outcomes, these base editors have enormous potential in biomedicine for correcting or introducing single point mutations. One example is CRISPR-STOP, a technique for truncating proteins by introducing stop codons in gene coding sequences using C>T base editing, offering an alternative method to knockout genes without relying on the unpredictable mutations resulting from introduction of DSBs [15].

Even though gene knockouts may be sufficient for eliminating certain proteins, they may not be ideal for gene therapies aiming to restore the natural state of healthy genomes, and an alternative strategy that can modulate the balance of different gene products may be more desirable than an on–off switch. This study demonstrates that single-base editors can be utilized to control gene splicing, a critical biological process by which pre-mRNA matures through removal of intronic sequences resulting in juxtaposition of exons to form mature transcripts prior to translation into proteins [16].

As the pre-mRNA transcript is processed, alternative splicing can result in some exons being excluded from the mature transcripts [16]. Alternative splicing provides temporal and tissue-specific control over which protein isoform is expressed and, therefore, plays a key role in biological complexity and development [16]. Importantly, synthetic regulation of alternative splicing provides critical molecular tools in biomedicine for selectively skipping mutation-containing exons from mature transcripts while keeping other normal isoforms intact [17].

## Methods

### Cell culture and transfection

The cell lines HCT116, 293T, MCF7, HEPG2, and Neuro-2A were obtained from the American Tissue Collection Center (ATCC). HCT116, 293T, and Neuro-2A cells were maintained in DMEM supplemented with 10% fetal bovine serum and 1% penicillin/streptomycin at 37 °C with 5% CO_2_. HEPG2 cells were maintained in DMEM supplemented with 10% fetal bovine serum, 1% penicillin/streptomycin, and 1% L-glutamine at 37 °C with 5% CO_2_. MCF7 cells were grown in EMEM supplemented with 10% fetal bovine serum, 1% penicillin/streptomycin, 0.1 mM non-essential amino acids, 1 mM sodium pyruvate, and 10 nM β-estradiol. All cell lines were transfected in 24-well plates with Lipofectamine 2000 (Invitrogen) following the manufacturer’s instructions. The amount of DNA used for lipofection was 1 μg per well. Transfection efficiency was routinely higher than 80% for 293T cells as determined by fluorescent microscopy following delivery of a control GFP expression plasmid. Transfection efficiency of other cell lines was lower (10–50%) and, therefore, we used puromycin selection for 48 h to enrich successfully transfected cells. Puromycin was used at a concentration of 1 μg/mL (HCT116, MCF7), 2 μg/mL (HepG2), or 3 μg/mL (Neuro2A).

### Plasmids and cloning

The plasmids used for SpCas9 sgRNA expression and expression of SpCas9, dCas9, and SpCas9-D10A were gifts from Charles Gersbach. The plasmids encoding SpCas9-BE3 (pCMV-BE3), SpCas9-VQR-BE3 (pBK-VQR-BE3), and SaCas9-KKH-BE3 (pJL-SaKKH-BE3) were gifts from David Liu (Addgene plasmids 73021, 85171, and 85170). The plasmid used for SaCas9-KKH-BE3 sgRNA expression (BPK2660) was a gift from Keith Joung (Addgene plasmid 70709). To facilitate enrichment of successfully transfected cells, we cloned a cassette for expression of puromycin N-acetyl-transferase and GFP tethered with T2A peptide from a PGK promoter into each of the three BE3 plasmids.

All oligonucleotides used in this work were obtained from IDT Technologies. The oligonucleotides for sgRNA generation were hybridized, phosphorylated and cloned into the appropriate sgRNA vector using BbsI sites for pSPgRNA and BsmBI sites for BPK2660 [18]. Guide sequences are provided in Additional file 1: Table S1.

### RT-PCR

RNA was harvested from cell pellets using the RNeasy Plus Mini Kit (Qiagen) according to the manufacturer’s instructions. cDNA synthesis was performed using the qScript cDNA Synthesis Kit (Quanta Biosciences) from 400 to 1000 ng of RNA with the cycling conditions recommended by the supplier. PCR was performed using KAPA2G Robust PCR kits from Kapa Biosystems. The 25 μL reactions used 50 ng of cDNA, Buffer A (5 μL), Enhancer (5 μL), dNTPs (0.5 μL), 10 μM forward primer (1.25 μL), 10 μM reverse primer (1.25 μL), KAPA2G Robust DNA Polymerase (0.5 U), and water (up to 25 μL). We used cycling parameters as recommended by the manufacturer. The PCR products were visualized in ethidium bromide-stained 2% agarose gels and images were captured using a ChemiDoc-It^2^ (UVP). The DNA sequences of the primers for each target are provided in Additional file 1: Table S2. PCR may favor shorter amplicons and introduce bias in the quantification of ratios of two transcripts of different lengths.

### Amplification of genomic DNA

Genomic DNA was isolated using the Animal Genomic DNA Purification Mini Kit (EarthOx). PCR was performed using KAPA2G Robust PCR kits (KAPA Biosystems) as described above, using 20–100 ng of template DNA.

### Deep sequencing

Deep sequencing was performed on PCR amplicons from genomic DNA or RNA harvested from duplicate transfections of 293T cells. After validating the quality of PCR product by gel electrophoresis, the PCR products were isolated by gel extraction using the Zymoclean Gel DNA Recovery Kit (Zymo Research). Shotgun libraries were prepared with the Hyper Library construction kit from Kapa Biosystems without shearing. The library was quantified by qPCR and sequenced on one MiSeq Nano flowcell for 251 cycles from each end of the fragments using a MiSeq 500-cycle sequencing kit version 2. Fastq files were generated and demultiplexed with the bcl2fastq v2.17.1.14 Conversion Software (Illumina). All sequencing was performed by the W. M. Keck Center for Comparative and Functional Genomics at the University of Illinois at Urbana-Champaign.

### Sequence analysis

Following sequence demultiplexing, genomic DNA reads were aligned with Bowtie2 [19]. To estimate base editing efficiency, base distribution was first calculated from the alignment, and duplicates were averaged. To determine statistically significant modification of intronic flanking G at the splice acceptor, *p*-values were calculated using a two-tailed Wald test assuming equal binomial proportions of G to non-G bases between control and base-edited samples. For the off-target analysis, a maximum likelihood estimate of 0.383% was obtained for the sequencing error rate of MiSeq by averaging the fraction of alternative allele depths calculated by SAMtools mpileup over all 90 on- and off-target sites in the control sample; significant G>A or C>T modifications at on- and off-target sites were then determined using the binomial test at a *p*-value cutoff of 10^− 5^, using the estimated sequencing error as the background probability of nucleotide conversions.

Reads from paired-end RNA-seq were mapped to the human genome version GRCh38 with TopHat2 [20] to determine the proportions of canonical and exon-skipped isoforms. Corresponding forward and reverse reads were then combined as one unit for counting analysis. Specifically, reads displaying an occurrence of the exon-skipped junction were counted towards the exon-skipped isoform, and reads displaying the canonical splice junction at the 5′ end of the exon to be skipped were contributed towards the canonical isoform. Reads that did not display either the exon-skipped junction or 5′ canonical splice junction of the exon to be skipped were discarded from quantification. A single estimate of the proportion and 95% confidence interval were obtained from the duplicates using the function “metaprop” from the R package “meta” with the inverse variance method to combine proportions and the Clopper-Pearson method to calculate the confidence interval. *P*-values for the RNA isoform quantification were also calculated using the two-tailed Wald test for equal binomial proportions between control and base-edited samples.

### Website design and genome-wide targetability analysis

The website scans all splice acceptor sites of the inner exons (those that are not the first or last exon of a transcript) of protein coding transcripts (genomic assembly GRCh38, GENCODE release 26) for PAMs in the appropriate range. The base editors supported are SaCas9-KKH-BE3, SpCas9-BE3, SpCas9-VRER-BE3, and SpCas9-VQR-BE3; only their primary PAMs (NNNRRT, NGG, NGCG, and NGA, respectively) were considered. The base editing efficiencies were estimated from the figures contained in Kim et al. [10]. The results of different experiments that reported editing efficiencies for the same position and base editor were averaged together. In order to minimize the number of false positives, a conservative estimate of the base-editing efficiency at each position was made by reporting a non-zero efficiency at a particular position only if the null hypothesis that the mean efficiency is negative was rejected with a *p*-value < 0.1 according to the *t*-test. To remove sgRNAs with potential off-targets, for each candidate sgRNA design, we scanned the genome for all sequences with at most two mismatches and calculated their off-target score [21]. We removed any sgRNA that has a top off-target score greater than 10.

## Results

**Fig. 1:**
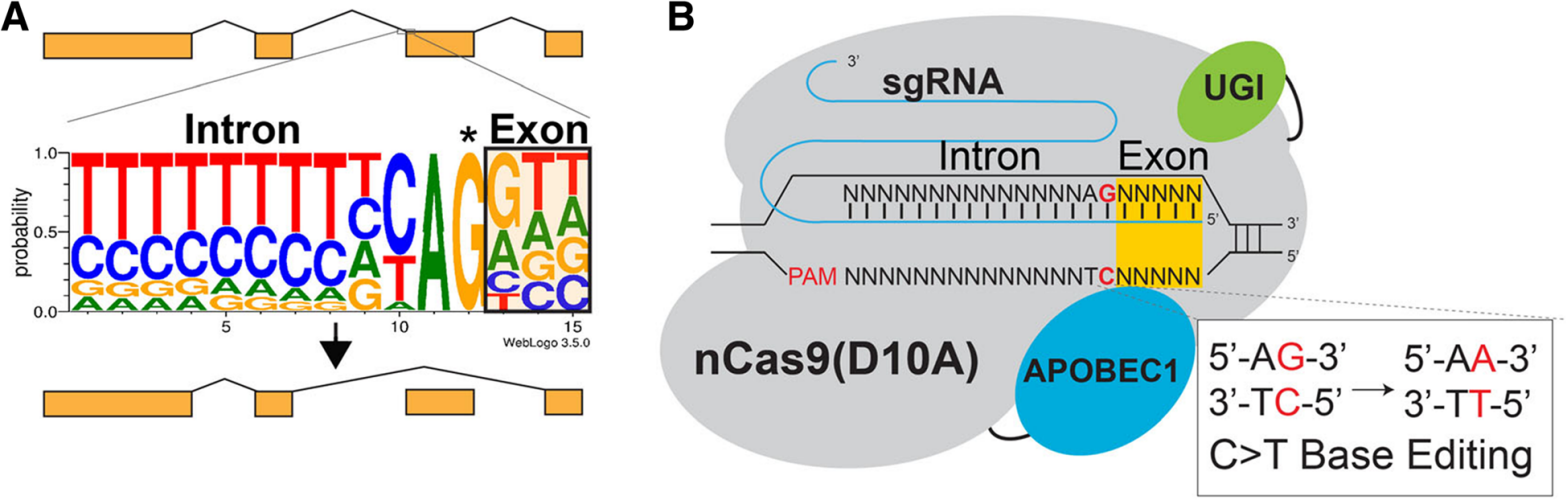
CRISPR-SKIP targeting strategy. **a** The consensus sequence of splice acceptors. We hypothesize that base editing of the highly conserved G (*asterisk*) leads to exon skipping. **b** In the presence of an appropriate PAM sequence, base editors can be utilized to deaminate the cytidine in the antisense strand, which is complementary to the conserved guanosine in the splice acceptor, thus resulting in the disruption of the splice acceptor and exon skipping

An essential step during exon splicing is the recognition by the spliceosome machinery of the highly conserved sequences that define exons and introns. More specifically, nearly every intron ends with a guanosine (Fig. 1a). For this reason, we hypothesized that mutations that disrupt this guanosine within the splice acceptor of any given exon in genomic DNA would lead to exon skipping by preventing incorporation of the exon into mature transcripts. Importantly, this guanosine can be effectively mutated by converting the complementary cytidine to thymidine using CRISPR-Cas9 C>T single-base editors [11], resulting in mutation of the target guanosine to adenosine and disruption of the highly conserved splice acceptor consensus sequence (Fig. 1b).

**Fig. 2:**
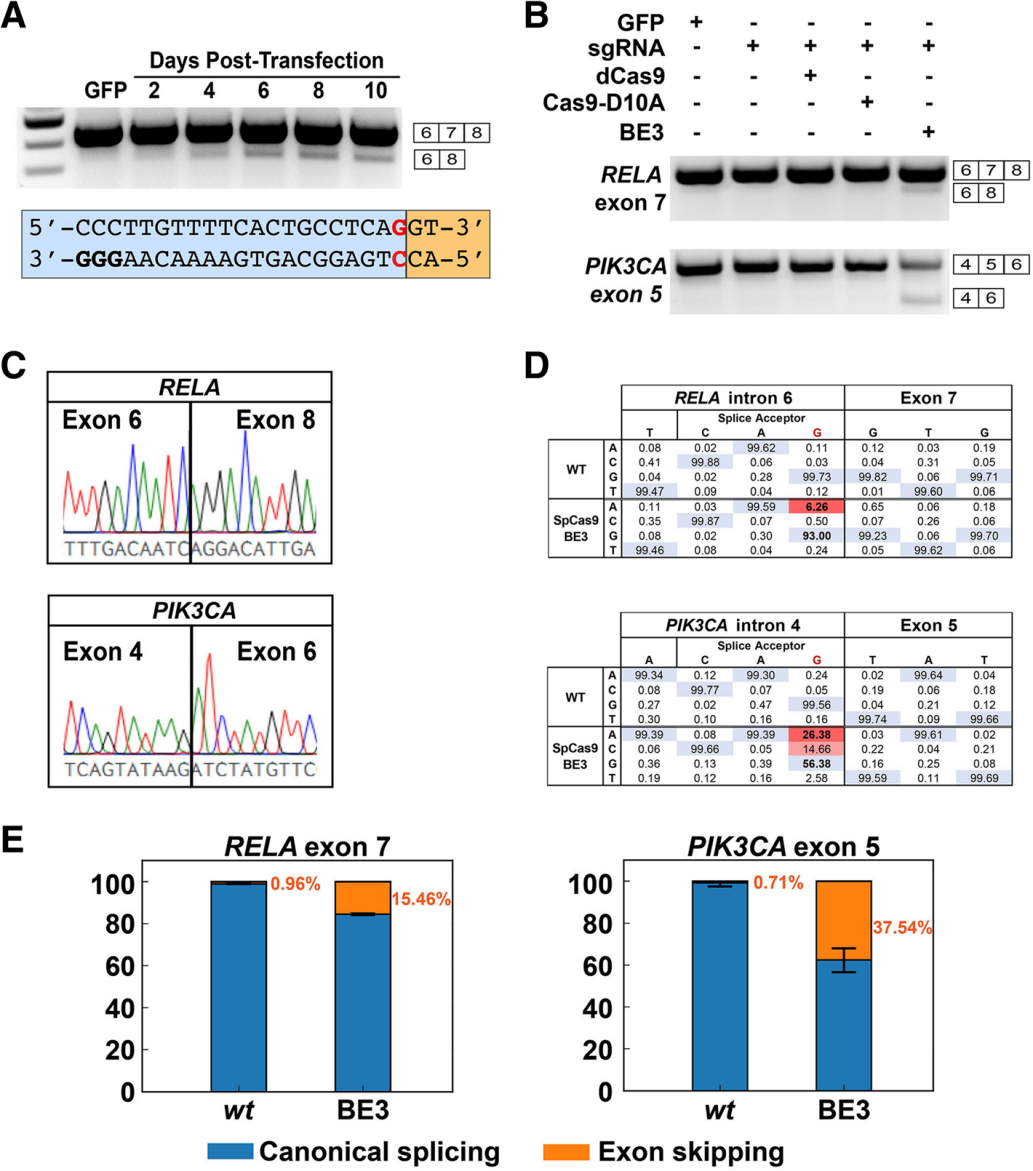
Single-base editing of splice acceptor consensus sequences enables programmable exon skipping. **a** 293T cells were transfected with C>T base editors and sgRNAs targeting the splice acceptor of exon 7 in *RELA*. RT-PCR was used to detect exon skipping over a 10-day time course. **b** Skipping of *RELA* exon 7 and *PIK3CA* exon 5 was induced by C>T base editors, but not by the sgRNA alone or in combination with dead SpCas9 or D10A nickase SpCas9. **c** Sanger sequencing of the exon-skipped amplicon was used to demonstrate successful exon skipping of *RELA* exon 7 and *PIK3CA* exon 5. **d** Deep sequencing of genomic DNA in wild-type (*WT*) cells and cells treated with C>T base editors targeting *RELA* exon 7 and *PIK3CA* exon 5 was used to calculate the modification rate. **e** Quantification of the rate of exon skipping of *RELA* exon 7 and *PIK3CA* exon 5 by deep sequencing of mature mRNA, which was amplified by RT-PCR

We first tested our hypothesis by inducing skipping of the 105 base pair (bp)-long exon 7 of *RELA*, a critical component of the NF-κB pathway implicated in inflammation and multiple types of cancer. We selected an exon whose length is a multiple of 3 to ensure that exon skipping would not create a frameshift, which could lead to nonsense-mediated decay and complicate the detection of novel splicing events. In these pilot experiments, we performed a time-course study in the embryonic kidney cell line 293T using the SpCas9-BE3 base editor [11], which is a combination of the rat APOBEC1 cytidine deaminase, the uracil glycosylase inhibitor of *Bacillus subtilis* bacteriophage PBS1, and the SpCas9-D10A nickase. As a derivative of SpCas9, this base editor recognizes target sites with an NGG proto-spacer adjacent motif (PAM), such as that existing upstream of *RELA* exon 7 (Fig. 2a). After transfecting SpCas9-BE3 and a sgRNA targeting the *RELA* exon 7 splice acceptor, we isolated RNA at different time points over a 10-day period, from which we prepared cDNA and analyzed exon skipping by PCR amplification. By gel electrophoresis, we observed that exon skipping is detectable for the first time 4 days after transfection, but the skipping frequency increases significantly on days 6, 8, and 10 (Fig. 2a). Based on these data we chose to analyze all subsequent experiments 6 days after transfection.

Next, we demonstrated that base editing of the splice acceptor was the mechanism underlying skipping of *RELA* exon 7 and *PIK3CA* exon 5, which could not be accomplished by transfection of the sgRNA alone or in combination with catalytically dead SpCas9 or SpCas9-D10A nickase (Fig. 2b). Importantly, Sanger sequencing confirmed the presence of transcripts with exon 6 followed by exon 8 in *RELA* and transcripts with exon 4 followed by exon 6 in *PIK3CA* (Fig. 2c). We quantified the efficiency of base-editing exon skipping in genomic DNA and cDNA using deep sequencing, which demonstrated that the G>A modification rates were 6.26% (*p* < 10^− 323^) for *RELA* and 26.38% (*p* < 10^− 323^) for *PIK3CA* (Fig. 2d, Additional file 2: Figure S1), leading to exon skipping rates in mRNA of 15.46% (*p* < 10^− 323^) for *RELA* and 37.54% (*p* = 7.38 × 10^− 37^) for *PIK3CA* (Fig. 2e). Interestingly, we also detected G>C (14.66%, *p* < 10^− 323^) and G>T (2.58%, *p* = 2.27 × 10^− 197^) editing events at *PIK3CA*. Furthermore, *PIK3CA* also exhibited an unexpected G>A modification (10.34%, *p* < 10^− 323^) outside the 20-nucleotide target sequence of the SpCas9-BE3 (Additional file 2: Figure S1).

**Fig. 3:**
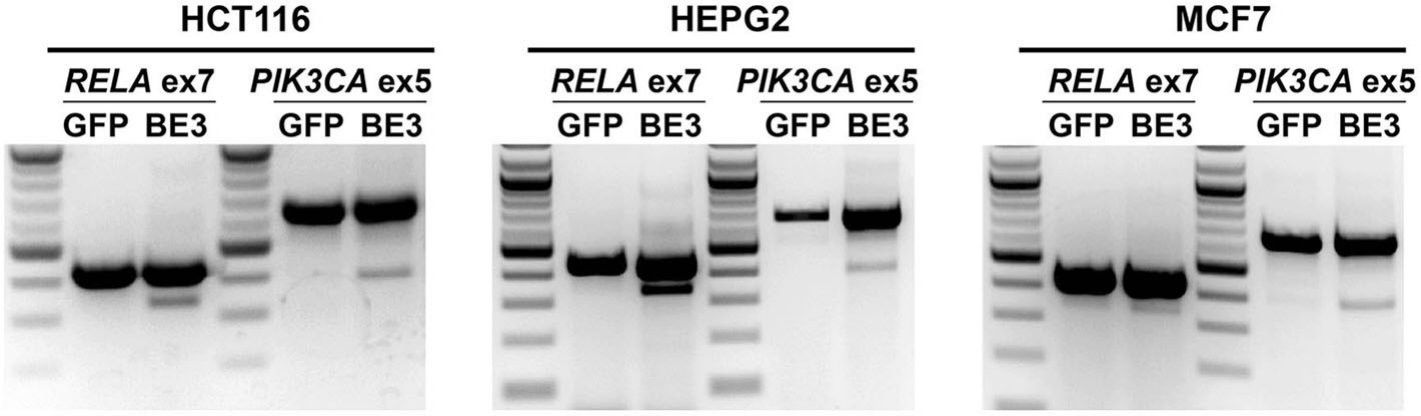
CRISPR-SKIP is effective across a panel of cell lines. CRISPR-SKIP induced skipping of *RELA* exon 7 and *PIK3CA* exon 5 in the cell lines HCT116, HEPG2, and MCF7

To determine whether our programmable exon skipping tools are cell line-specific, we targeted the same two exons in the human cell lines HCT116, HepG2, and MCF7, as well as *RELA* exon 8 in the mouse cell line Neuro-2A (Fig. 3, Additional file 2: Figure S2). Since the transfection efficiency in these cell lines is typically lower than that in 293T cells, we enriched for transfected cells prior to analysis, which revealed successful skipping of the targeted exon in all cell lines tested.

**Fig. 4:**
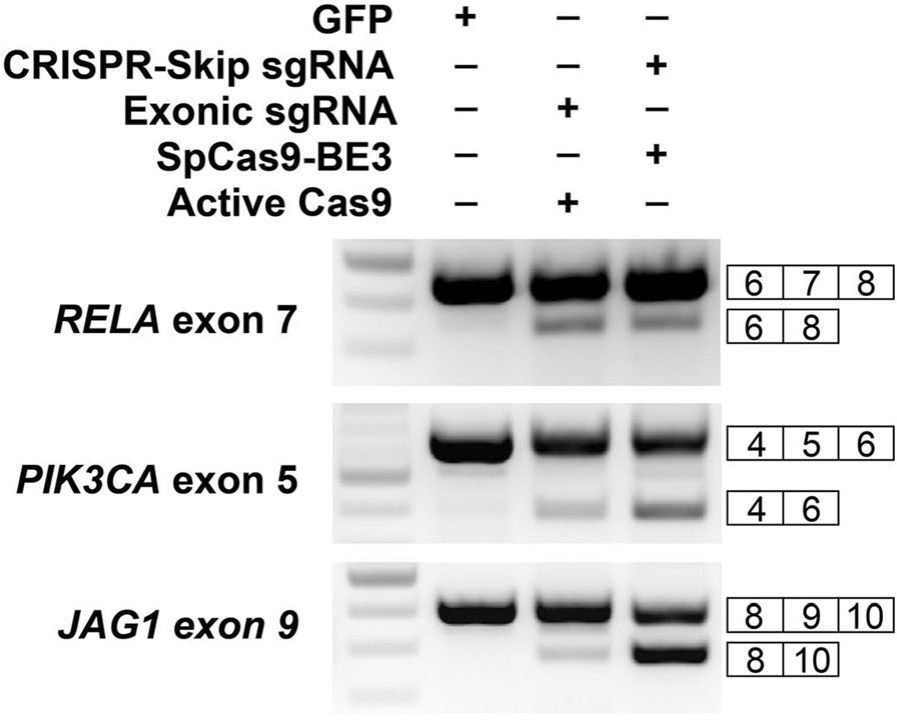
Comparison of CRISPR-SKIP with active SpCas9 for inducing exon skipping. CRISPR-SKIP was utilized to target the splice acceptors of *RELA* exon 7, *PIK3CA* exon 5, and *JAG1* exon 9. In parallel, sgRNAs targeting the same exons were co-transfected with active SpCas9 to induce exon skipping. Analysis by PCR demonstrates that CRISPR-SKIP induced exon skipping at equal or greater rates than active SpCas9 in each of three exons tested

We sought to compare CRISPR-SKIP to current state-of-the-art exon skipping using gene editing, which relies on introduction of DSBs to generate random repair outcomes, some of which cause exon skipping [22]. We employed CRISPR-SKIP and, separately, targeted active SpCas9 to the exons of *RELA* exon 7, *PIK3CA* exon 5, and *JAG1* exon 9. In each case, we achieved an equal or greater degree of exon skipping with CRISPR-SKIP than with active SpCas9 (Fig. 4). Since introduction of DSBs in the exon required sgRNAs different from those used to target the splice acceptor with CRISPR-SKIP, the comparison of these two techniques might be biased towards that using the more efficient sgRNAs. For this reason, we also performed a comparison of exon skipping by active SpCas9 and CRISPR-SKIP using identical sgRNAs targeting the splice acceptor across five different targets. In these conditions, active SpCas9 induced higher rates of exon skipping at three targets, while CRISPR-SKIP was more effective at two targets. Active SpCas9 induced exon skipping at all targets tested, while CRISPR-SKIP was effective at four out of five targets (Additional file 2: Figure S3).

**Fig. 5:**
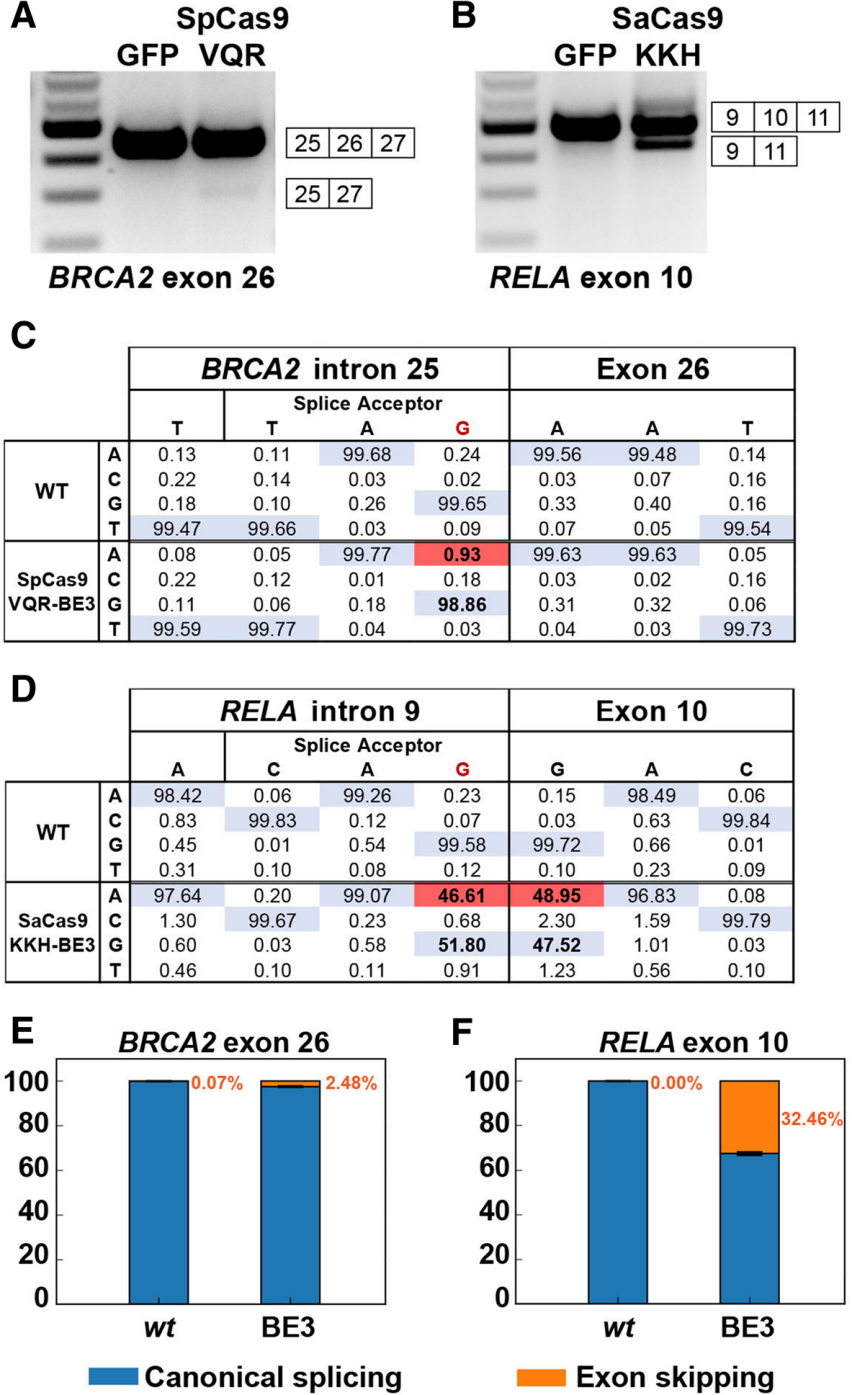
Different Cas9 scaffolds increase the number of CRISPR-SKIP target exons. **a, b** RT-PCR analysis demonstrates that SpCas9-VQR-BE3 (**a**) and SaCas9-KKH-BE3 (**b**) can induce exon skipping of *BRCA2* exon 26 and *RELA* exon 10, respectively. **c, d** Deep sequencing of genomic DNA revealed that targeted mutations (*red*) introduced by SpCas9-VQR-BE3 were found in 0.93% of reads at the *BRCA2* exon 26 splice acceptor (**c**), while SaCas9-KKH-BE3 induced targeted mutations in 46.61% of reads at *RELA* exon 10 splice acceptor (**d**). Deep sequencing was performed in biological duplicates, and the results were combined. **e, f** Quantification of the rate of exon skipping of *BRCA2* exon 26 (**e**) and *RELA* exon 10 (**f**) by deep sequencing of mature mRNA, which was amplified by RT-PCR. RNAseq was performed on biological duplicates and a single estimate of the proportion and confidence intervals were obtained (“Methods”)

One limitation of CRISPR-SKIP using SpCas9-BE3 is its dependence on the presence of a PAM site located 12–17 bp from the target cytidine. SpCas9-BE3 canonically recognizes NGG PAMs, but can also recognize NAG with lower efficiency and both can be used for skipping target exons (Additional file 1: Table S1). However, not all exons have one of the SpCas9-BE3 PAMs within the desired range. To expand the number of targetable exons, we also demonstrated that single-base editors constructed using different Cas9 scaffolds [10], which recognize different PAM motifs, can be used in CRISPR-SKIP. Specifically, we successfully used the SpCas9-VQR-BE3, which recognizes NGA PAMs, to skip exon 26 in the *BRCA2* gene (Fig. 5a) and the SaCas9-KKH-BE3 editor, which recognizes NNNRRT PAMs, to skip exon 10 in *RELA* (Fig. 5b). Deep sequencing of SpCas9-VQR-BE3 and SaCas9-KKH-BE3 edited cells revealed targeted G>A modification rates of 0.93% (*p* = 4.74 × 10^− 47^) by SpCas9-VQR-BE3 at *BRCA2* exon 26 (Fig. 5c) and 46.61% (*p* < 10^− 323^) by SaCas9-KKH-BE3 at *RELA* exon 10 (Fig. 5d). Interestingly, the first base in *RELA* exon 10, a guanosine within the optimal target range for SaCas9-KKH-BE3, was modified in 48.95% (*p* < 10^− 323^) of the DNA strands (Fig. 5d, Additional file 2: Figure S4). At this target, the exonic base was modified without modifying the intronic base in only 2.9% of the reads, whereas the intronic base was modified without modifying the exonic base in only 0.7% of the reads. Targeted deep sequencing of cDNA was performed on CRISPR-SKIP-treated cells to quantify exon skipping events. CRISPR-SKIP resulted in 2.48% (*p* = 1.33 × 10^− 172^) skipping rate in *BRCA2* exon 26 (Fig. 5e) and 32.46% (*p* < 10^− 323^) skipping rate in *RELA* exon 10 (Fig. 5f).

Cas9 can bind DNA even when the sgRNA is not perfectly matched, which can result in undesired modifications in the genome. To assess the extent of off-target effects, we targeted CRISPR-SKIP to 16 exons using 18 sgRNAs and sequenced the genomic DNA at on-target sites as well as four high scoring [21] off-target sites for each sgRNA. We found that 14 out of 18 (77.78%) sgRNAs successfully modified their respective on-target sites, while only 10 out of 72 (13.89%) predicted off-target sites showed evidence of modification (Table 1).

**Table 1:**
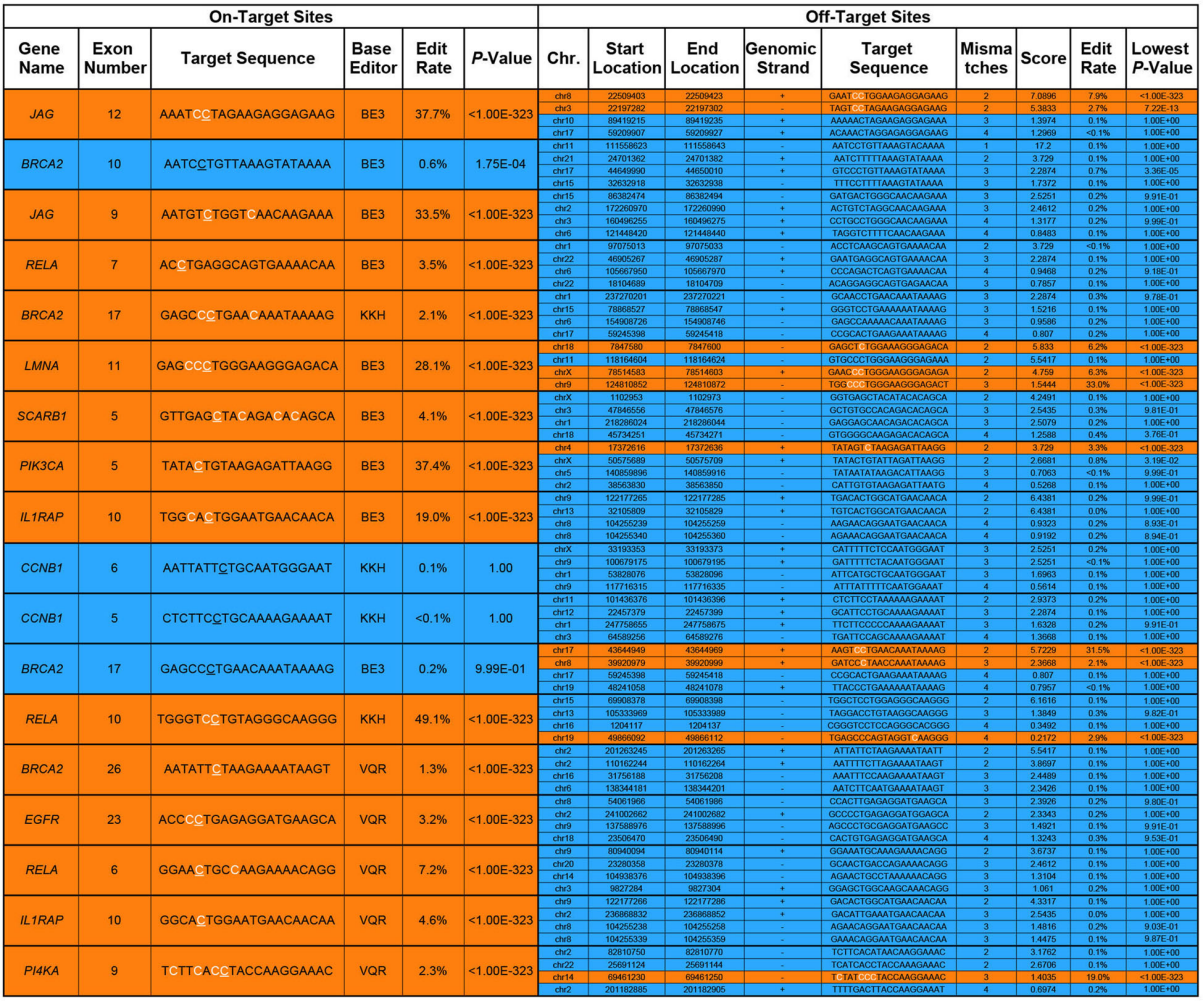
Summary of off-target modification analysis by next-generation sequencing

*Left column* displays on-target sites each with four corresponding off-target sites in the *right column*. Sites displaying statistically significant C>T or G>A conversion are colored *orange*, while sites not displaying such conversion are colored *blue*. The specific locations in the target sequences where statistically significant conversion was observed are colored *white*. For the on-target site target sequences, the base that corresponds to the flanking intronic G is *underlined*. The “Edit rate” and *“P*-value” columns in the on-target section refer to the flanking intronic G. The “Edit rate” and “Lowest *P*-value” columns in the off-target section refer to the location within the off-target sequence with the most statistically significant C>T or G>A conversion

**Fig. 6:**
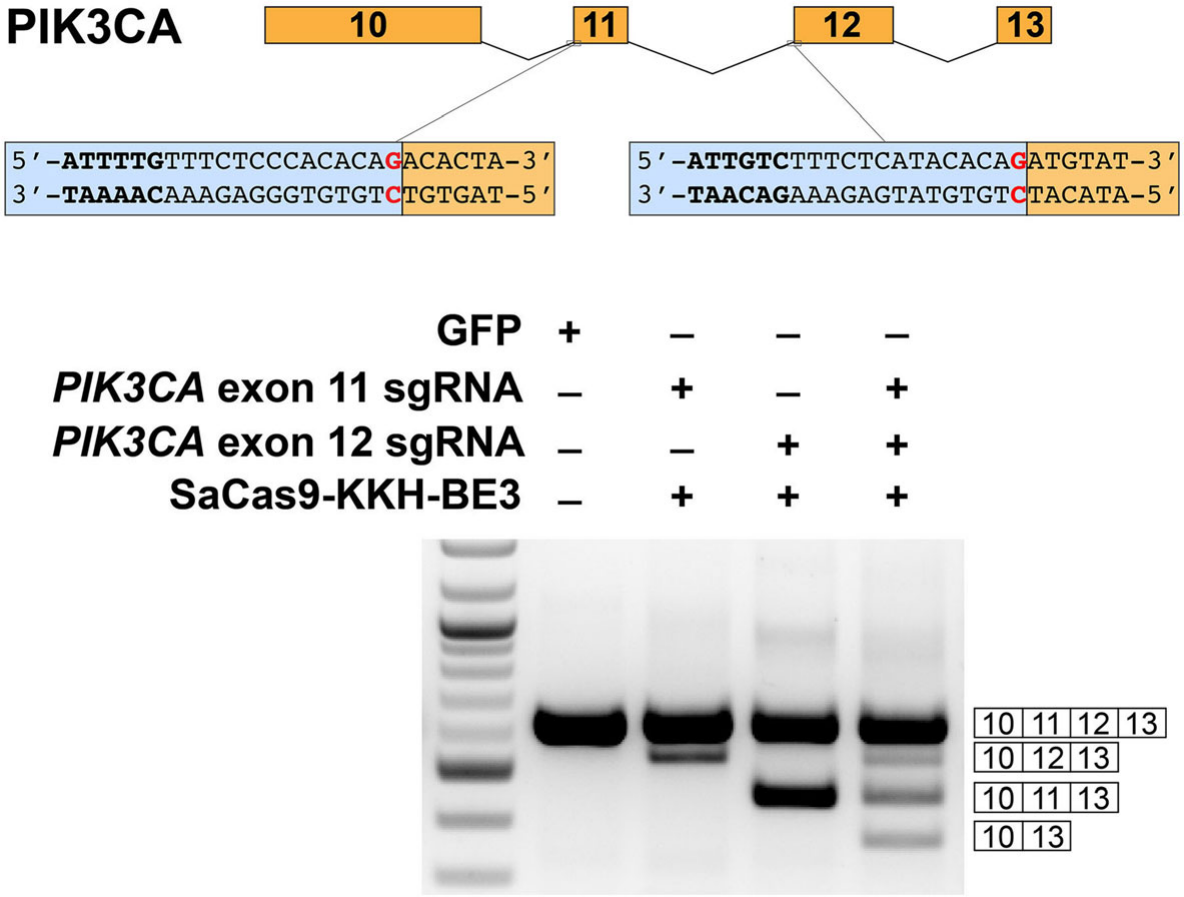
CRISPR-SKIP can be used to simultaneously skip multiple exons within the same transcript. SaCas9-KKH-BE3 was used to target *PIK3CA* exons 11 and 12. RT-PCR demonstrated that both sgRNAs induced skipping of the targeted exon and, when used together, induced skipping of both exons simultaneously

Therapeutic exon skipping often requires inducing splicing of multiple exons simultaneously within the same transcript to recover a reading frame [17]. Since CRISPR base-editing tools are theoretically capable of multiplexing, but this property has not been conclusively demonstrated previously in human cells, we tested whether CRISPR-SKIP could induce simultaneous skipping of two exons by targeting *PIK3CA* exons 11 and 12. Analysis by RT-PCR revealed that SpCas9-BE3 editing tools can successfully induce skipping of *PIK3CA* exons 11 or 12 when used individually; when combined, they induce skipping of exon 11, exon 12, and both exons 11 and 12 (Fig. 6).

**Fig. 7:**
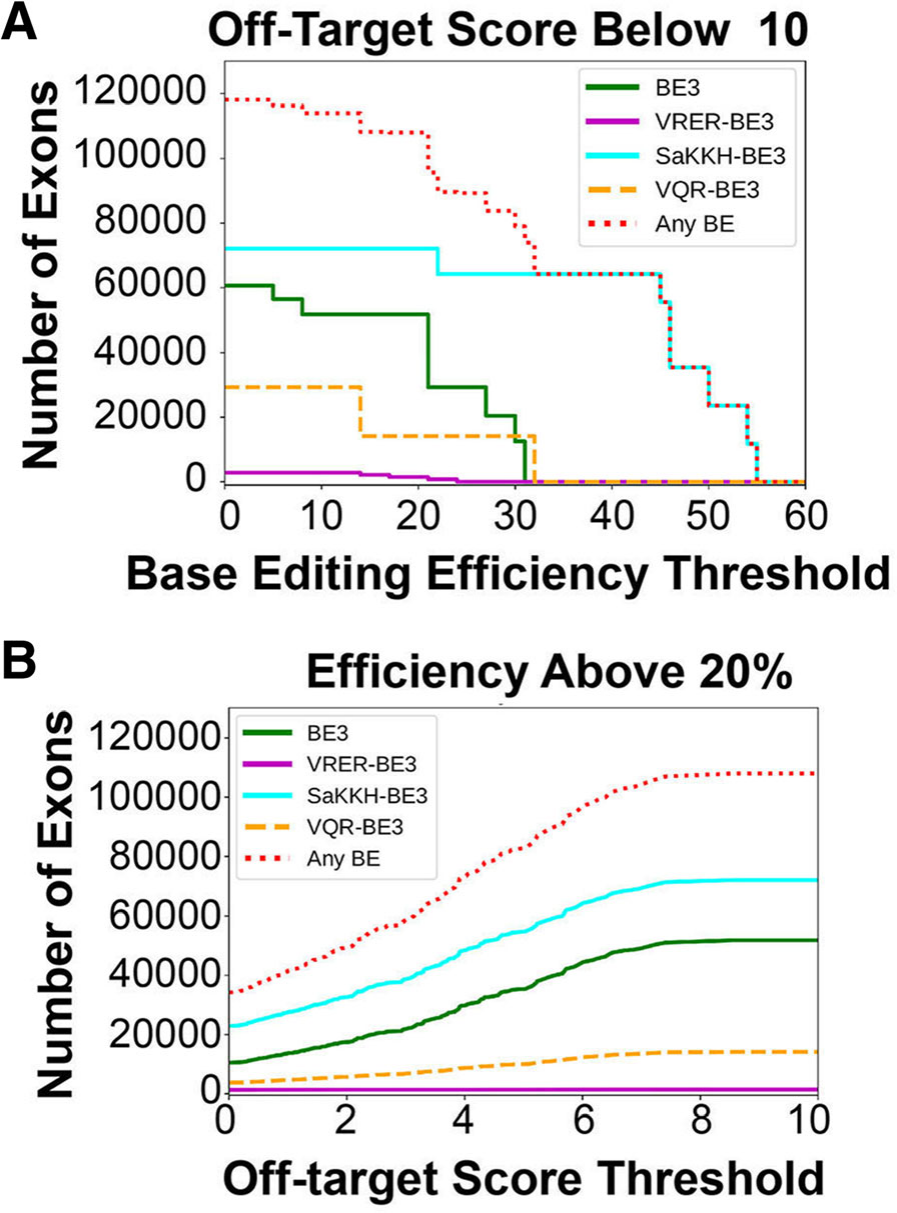
Genome-wide computational estimation of targetability by CRISPR-SKIP. **a** Estimation of the number of exons that can be targeted by each base editor with estimated efficiency of editing flanking intronic G at or above the corresponding value on the *x*-axis. Only exons with maximum off-target score below 10 are considered. **b** Estimation of the number of exons that can be targeted by each base editor with maximum off-target score at or below the corresponding value on the *x*-axis. Only exons for which the estimated efficiency of editing the flanking G nucleotide is above 20% are considered

To facilitate the identification of exons that can be skipped with the various base editors, we developed a web-based software tool that enables rapid identification of potential CRISPR-SKIP sgRNAs given a desired target gene or exon (http://song.igb.illinois.edu/crispr-skip/). The software incorporates the known base-editing efficiency profiles of the base editors SpCas9-BE3, SaCas9-KKH-BE3, SpCas9-VQR-BE3, and SpCas9-VRER-BE3 [10]. We estimate that these four base editors together enable targeting of 118,089 out of 187,636 inner exons in protein coding transcripts (genome assembly version GRCh38 and GENCODE release 26) at the off-target score [21] cutoff of 10, where 100 corresponds to perfect matching on targets (Fig. 7, Additional file 2: Figure S5, Additional file 2: Figure S6, “Methods”).

## Discussion

CRISPR-SKIP is a method for controlling isoform-specific gene expression with diverse research applications in biology and biotechnology. For example, it may enable the study of alternatively spliced genes whose various protein isoforms have distinct roles in tissue specification and development [23]. CRISPR-SKIP could also be utilized to study the function of long non-coding RNAs (lncRNAs) consisting of multiple exons that are spliced in much the same way as protein-coding RNA transcripts [24]. As lncRNAs do not encode a protein, knockout strategies that incorporate premature STOP codons cannot be applied to perturb lncRNA levels. Furthermore, interrogating lncRNA by transcriptional silencing is complex, because their promoters are frequently multi-functional and regulate expression of multiple elements [25]. By contrast, CRISPR-SKIP provides a method for identifying and excluding functional domains from lncRNAs with a level of precision that no other gene-editing technique can achieve.

Importantly, CRISPR-SKIP also has multiple potential applications in biomedicine, given that exon skipping strategies have already shown promise for treating several monogenic diseases, such as Leber congenital amaurosis [26], atherosclerosis [27], FTDP-17 [28], cancer [29], rheumatoid arthritis [30], Huntington’s disease [17], dystrophic epidermis bullosa [31], and Duchenne muscular dystrophy (DMD) [32]. Exon skipping is especially exciting for the treatment of DMD, as targeting one or two exons could ameliorate the effects of 79% of DMD deletions and 91% of DMD small mutations, for a total of 77% of all DMD mutations [33]. Our data suggest that CRISPR-SKIP can produce exon skipping at therapeutically significant levels. In the treatment of DMD, as little as 4% recovery of dystrophin [34, 35] restores significant muscle function. Similarly, a 40% reduction of mutant Huntingtin is sufficient for clinical improvement in models of Huntington’s disease. In addition to recovering the reading frame of mutant genes, CRISPR-SKIP allows for isoform-specific modulation that cannot be achieved by introducing premature stop codons through current gene-editing strategies [26–28].

To date, techniques for targeted exon skipping are either transient, such as injection of antisense oligonucleotides [17], or require introduction of DSBs into coding and/or non-coding regions of the genome, which could lead to deleterious off-target effects [22, 36]. In this manuscript we characterized CRISPR-SKIP, a technology that induces permanent modifications in the genome without DSBs, thus providing a significant advantage over other exon skipping techniques. Since the changes introduced by CRISPR-SKIP are hardwired in the genome after a single treatment, this technology is especially attractive as a potential therapeutic tool for a wide variety of human diseases.

It is noteworthy that in our experiments we achieved statistically significant base editing at 77.78% of splice acceptor sites we targeted, but we were able to skip only 50% of the corresponding exons (56% with SpCas9-BE3, 50% with SaCas9-BE3, and 40% with SpCas9-VQR-BE3; Table 1, Additional file 1: Table S1). Five targets, *JAG1* exon 12, *BRCA2* exon 17 (when targeted with SaCas9-KKH-BE3), *EGFR* exon 23, *LMNA* exon 11, and *RELA* exon 6, exhibited statistically significant base editing, but no exon skipping by RT-PCR. Detailed analysis of each of these exons revealed the presence of cryptic splice acceptor sites, which may have been activated when the native site was destroyed. While the reason why base editors fail to induce skipping of some exons remains unknown, we anticipate that improved understanding of exon–intron architecture and their recognition by the spliceosome machinery will enable more efficient targeting in the future. Similarly, advancements in base-editing technologies will likely improve the rate of exon skipping as well as span the number of exons that can be effectively targeted. For example, the recently described xCas9-BE3 [37], which enables editing of protospacers with NG, GAA, and GAT PAMs, is predicted to broaden the targeting range of CRISPR-SKIP.

Interestingly, in the samples in which the target exon was successfully skipped, we observed some discrepancies between genomic DNA editing efficiency and the measured rate of exon skipping, which could be explained by the lower number of splice events that an exon-skipped transcript must undergo. In fact, one of the major blocks during transcript elongation is the splicing junction [38–40], as demonstrated by the findings that splicing leads to transient polymerase pausing at the splice sites [41].

We also characterized off-target modifications introduced by CRISPR-SKIP and even though only 13.9% of tested off-target sites were actually modified, these sites corresponded to 6 out of 18 (33.3%) distinct sgRNAs. Only three of these mutations occurred in coding sequences, but it will be important for future applications of CRISPR-SKIP to mitigate off-target effects by using newer generations of base editors. More specifically, target specificity can be increased through several recent additions to the base-editing toolkit, such as high fidelity base editors that have decreased affinity for genomic DNA and thus rely on longer sequences of sgRNA–DNA base complementarity for binding [42]. Another example is the base editor BE4-GAM, which, by decreasing indels introduced by Cas9 nickase, has been shown to reduce unintended mutations [43]. However, we have observed that the exon-skipping activity of BE4-GAM is lower than the activity of BE3 at some target sites (Additional file 2: Figure S7); therefore, it is important to test various base editors to identify a proper balance between activity and specificity. Our off-target analysis also supports that off-target effects are mostly derived from off-target Cas9 binding, indicating that high-fidelity base editors [42] may effectively decrease CRISPR-SKIP off-target mutations while preserving activity.

The importance of selecting a proper gene-editing tool is highlighted by the finding that when the splice acceptor is immediately followed by a G at the 3′ end, the base editor may introduce a G>A mutation in the bystander exonic base without modifying the splice acceptor. When the purpose of the experiment is to skip an exon already containing a mutation, the impact of this bystander mutation will likely be minimal. For applications in which this mutation is unacceptable, there are several alternative approaches to shift the editing window. For example, when additional PAM sites are available, a different sgRNA can be used to force the exonic base out of the editing window (note that *RELA* exon 7 begins with a G, in sgRNA position 2, which undergoes minimal editing). When this is not an option, Cas9 variants that have been engineered specifically for editing within narrow windows can be used [10]. Finally, the linker connecting the Cas9 scaffold and the cytidine deaminase plays a critical role defining the cytidine that is modified as well as the modification rate [11, 43]. Therefore, it is possible that optimization of the domain structure of base editors may prevent bystander mutations.

Finally, our results indicate that CRISPR-SKIP efficiency at inducing exon skipping is higher than the efficiencies of gene-editing methods that introduce DSBs in coding sequences and similar to those of methods that introduce DSBs near the splice acceptor [37]. In terms of specificity, however, it is important to note that the stochasticity of DSB repair, as well as the potential for translocations and other chromosomal aberrations that are not typically detected by current methods for analyzing off-target modifications, renders active Cas9 less predictable and potentially less safe than CRISPR-SKIP.

## Conclusions

The results presented in this manuscript demonstrate that programmable exon skipping can be accomplished by disrupting splice acceptors using single-base editors. One major advantage of CRISPR-SKIP over other methods is that it introduces changes in the genome that are permanent without requiring DSBs to alter genomic DNA. Given the current availability of various base editors that use different Cas9 scaffolds, we estimate that 118,089 out of 187,636 inner exons in protein coding transcripts can be targeted. We demonstrated that this method is multiplexable, is applicable to multiple cell lines of diverse species, and can achieve skipping rates as high as 32.46%. Our study also provides a webtool for rapidly identifying and designing potential target sites in the entire human genome.

## Additional files

Additional file 1: **Tables S1 and S2.** Supplemental tables. (PDF 4027 kb)

Additional file 2: **Figures S1–S7.** Supplemental figures. (PDF 187 kb)
