# Supplementary material for: CRISPR-SKIP: programmable gene splicing with single base editors
Source: Genome Biol. 2018 Aug 15;19:107. doi: 10.1186/s13059-018-1482-5 (PMC6092781; doi:10.1186/s13059-018-1482-5)
Supplement: Supplementary file 1 — Tables S1 and S2. Supplemental tables. (PDF 4027 kb) [file 13059_2018_1482_MOESM1_ESM.pdf]

| Target Gene | Target Exon Number | Ensembl ID: Exon Number | BE3 Version | Target Sequence | PAM | Exon Skipped? (by gel electrophoresis) |
|---|---|---|---|---|---|---|
| BRCA2 | 10 | ENST00000380152.7:10 | SpBE3 | AATCCTGTTAAAGTATAAAA | CAG | Y |
| BRCA2 | 17 | ENST00000380152.7:17 | SpBE3 | GAGCCCTGAACAAATAAAAG | TAG | N |
| BRCA2 | 17 | ENST00000380152.7:17 | SaBE3-KKH | GAGCCCTGAACAAATAAAAG | TAGAAT | N |
| BRCA2 | 26 | ENST00000380152.7:26 | SpBE3-VQR | AATATTCTAAGAAAATAAGT | GGA | Y |
| CCNB1 | 5 | ENST00000256442.9:5 | SaBE3-KKH | CTCTTCCTGCAAAAGAAAAT | GCTGAT | N |
| CCNB1 | 6 | ENST00000256442.9:6 | SaBE3-KKH | AATTATTCTGCAATGGGAAT | TTCAAT | N |
| EGFR | 23 | ENST00000275493.6:23 | SpBE3-VQR | ACCCCTGAGAGGATGAAGCA | AGA | N |
| IL1RAP | 10 | ENST00000447382.5:10 | SpBE3-VQR | GGCACTGGAATGAACAACAA | AGA | Y |
| IL1RAP | 10 | ENST00000447382.5:10 | SpBE3 | TGGCACTGGAATGAACAACA | AAG | N |
| JAG1 | 9 | ENST00000254958.9:9 | SpBE3 | AATGTCTGGTCAACAAGAAA | AGG | Y |
| JAG1 | 12 | ENST00000254958.9:12 | SpBE3 | AAATCCTAGAAGAGGAGAAG | GGG | N |
| LMNA | 11 | ENST00000448611.6:11 | SpBE3 | GAGCCCTGGGAAGGGAGACA | AGG | N |
| PI4KA | 9 | ENST00000255882.10:9 | SpBE3-VQR | TCTTCACCTACCAAGGAAAC | AGA | N |
| PIK3CA | 5 | ENST00000263967.3:5 | SpBE3 | TATACTGTAAGAGATTAAGG | GGG | Y |
| PIK3CA | 11 | ENST00000263967.3:11 | SaBE3-KKH | TAGTGTCTGTGTGGGAGAAA | CAAAAT | Y |
| PIK3CA | 12 | ENST00000263967.3:12 | SaBE3-KKH | ATACATCTGTGTATGAGAAA | GACAAT | Y |
| RELA | 6 | ENST00000406246.7:6 | SpBE3-VQR | GGAACTGCCAAGAAAACAGG | CGA | N |
| RELA | 7 | ENST00000406246.7:7 | SpBE3 | ACCTGAGGCAGTGAAAACAA | GGG | Y |
| RELA | 10 | ENST00000406246.7:10 | SaBE3-KKH | TGGGTCCTGTAGGGCAAGGG | CTAGGT | Y |
| SCARB1 | 5 | ENST00000339570.9:5 | SpBE3 | GTTGAGCTACAGACACAGCA | GGG | Y |

**Table S1.** Results of CRISPR-SKIP targeting at 18 human sites with 20 sgRNAs. Target base is shown in red.

| Designation | Target Gene | Target Exon | Primer Sequence | RT PCR or |
|---|---|---|---|---|
| BRCA2int9 F | BRCA2 | 10 | AACAGGAGAAGGGGTGACTGAC | gDNA |
| BRCA2ex10 R | BRCA2 | 10 | TTCCAATGTGGTCTTTGCAGCT | gDNA |
| BRCA2ex7 F | BRCA2 | 10 | GCTACACCACCCACCCTTAGTT | RT |
| BRCA2ex11 R | BRCA2 | 10 | TTCCTGCAGGCATGACAGAGAA | RT |
| BRCA2int16 F | BRCA2 | 17 | agatgtgggggtctcactatgttg | gDNA |
| BRCA2ex17 R | BRCA2 | 17 | AGCTGCCAGTTTCCATATGATCCA | gDNA |
| BRCA2ex15 F | BRCA2 | 17 | CACAGCCAGGCAGTCTGTATCT | RT |
| BRCA2ex18 R | BRCA2 | 17 | TGGGGCTTCAAGAGGTGTACAG | RT |
| BRCA2int25 F | BRCA2 | 26 | aggacttgagccccaatcttcc | gDNA |
| BRCA2ex26 R | BRCA2 | 26 | GTGTACGGCCCTGAAGTACAGT | gDNA |
| BRCA2ex25 F | BRCA2 | 26 | TCTGCTAGTCCAAAAGAGGGCC | RT |
| BRCA2ex27 R | BRCA2 | 26 | CTGTGCAGCCGGAGAAACAAAT | RT |
| CCNB1int4 F | CCNB1 | 5 | aagcaatctgccaacttcagcc | gDNA |
| CCNB1ex5 R | CCNB1 | 5 | CAGTGACTTCCCGACCCAGTAG | gDNA |
| CCNB1int5 F | CCNB1 | 6 | CCCTTCCAGGATTCTAGCCGAG | gDNA |
| CCNB1ex6 R | CCNB1 | 6 | AAACATGGCAGTGACACCAACC | gDNA |
| CCNB1ex3 F | CCNB1 | 5 and 6 | gagccagaacctgagccTGTTA | RT |
| CCNB1ex7 R | CCNB1 | 5 and 6 | AGGAGGAAAGTGCACCATGTCA | RT |
| EGFRint22 F | EGFR | 23 | gaggtagactgaggcttccagc | gDNA |
| EGFRint23 R | EGFR | 23 | GATGCAAAGGCCTCAGCTGTTT | gDNA |
| EGFRex20 F | EGFR | 23 | GCTCAACTGGTGTGTGCAGATC | RT |
| EGFRex24 R | EGFR | 23 | TCACGGAACTTTGGGCGACTAT | RT |
| IL1RAPint9 F | IL1RAP | 10 | accgtggacttcttcaggtagc | gDNA |
| IL1RAPex10 R | IL1RAP | 10 | GCTCCAAAACCACAAGCCAGTT | gDNA |
| IL1RAPex8 F | IL1RAP | 10 | CTCGCAATGAGGTTTGGTGGAC | RT |
| IL1RAPex11.12 R | IL1RAP | 10 | GTATTTCCCCCAGGCAGACTGT | RT |
| JAG1int8 F | JAG1 | 9 | CTTGTAGCAGGTGTCTGGCTCT | gDNA |
| JAG1ex9 R | JAG1 | 9 | GGGGCACACACACTTAAATCCG | gDNA |
| JAG1ex8 F | JAG1 | 9 | GAGGCAGCTGTAAGGAGACCTC | RT |
| JAG1ex12 R | JAG1 | 9 | CTGCATAGCCAGGTGGACAGAT | RT |
| JAG1ex7 F | JAG1 | 9 long range | GAACTTGTAGCAACACAGGCCC | RT |
| JAG1ex15 R | JAG1 | 9 long range | AGGAGTTGACACCATCGATGCA | RT |
| JAG1int11 F | JAG1 | 12 | AGCTAAACCGCAACAGTCATGC | gDNA |
| JAG1int12 R | JAG1 | 12 | CCATCTGAGGTTTTGCCACCAC | gDNA |
| JAG1ex9 F | JAG1 | 12 | CGGATTTAAGTGTGTGTGCCCC | RT |
| JAG1ex13 R | JAG1 | 12 | TTCTGGCAGGGATTAGGCTCAC | RT |
| LMNAint10 F | LMNA | 11 | AAGCTTGCTCCCGTTCTCTCTT | gDNA |
| LMNAint11 R | LMNA | 11 | CAGAAGAGCCAGAGGAGATGGG | gDNA |
| LMNAex10 F | LMNA | 11 | GACGACGAGGATGAGGATGGAG | RT |
| LMNAex12 R | LMNA | 11 | CACCCCTTTCCCTTGGCTTCTA | RT |
| PI4KAint8 F | PI4KA | 9 | CTGAGGTCTGCACATCCTGGAA | gDNA |
| PI4KAint9 R | PI4KA | 9 | CACTGCAAAACCCCTTCCACTC | gDNA |
| PI4KAex8 F | PI4KA | 9 | ACTGCCCTAGAGCCTGAGTACT | RT |
| PI4KAex10 R | PI4KA | 9 | CACGCAGCATCTTGAACATGGT | RT |

| | | | | |
|---|---|---|---|---|
| PI4KAex31 F | PI4KA | 33 | CCATGTTCAAGCTGACCGCAAT | RT |
| PI4KAex36 R | PI4KA | 33 | GCTGGCGGTCAGGTACTTCTTA | RT |
| PIK3CAint4 F | PIK3CA | 5 | ggggtttcaccgttttagccag | gDNA |
| PIK3CAex5 R | PIK3CA | 5 | ACATCAAATTGGGCATCCTCCC | gDNA |
| PIK3CAex3 F | PIK3CA | 5 | TCTTCACCAGAATTGCCAAAGC | RT |
| PIK3CAex6 R | PIK3CA | 5 | TCCACCTGGGATTGGAACAAGG | RT |
| PIK3CAex2 F | PIK3CA | 5 long range | ACCAGTAGGCAACCGTGAAGAA | RT |
| PIK3CAex9 R | PIK3CA | 5 long range | TCGGGATACAGACCAATTGGCA | RT |
| PIK3CAex9 F | PIK3CA | 11 and 12 | TAGCTATTCCCACGCAGGACTG | RT |
| PIK3CAex14 R | PIK3CA | 11 and 12 | TTCCATTGCCTCGACTTGCCTA | RT |
| RELAint5 F | RELA | 6 | TTTCCTGCATCTCCCTCACTGG | gDNA |
| RELAex6 R | RELA | 6 | ACAGCATTCAGGTCGTAGTCCC | gDNA |
| RELAint6 F | RELA | 7 | CAGATTGGCAcccactggacta | gDNA |
| RELAex7 R | RELA | 7 | AGATCTTGAGCTCGGCAGTGTT | gDNA |
| RELAex8 R | RELA | 6 and 7 | CCTGGTCCCGTGAAATACACCT | RT |
| RELAex4 F | RELA | 6 and 7, and 7 long | CCCACGAGCTTGTAGGAAAGGA | RT |
| RELAex11 R | RELA | 7 long range | TGCTCAGGGATGACGTAAAGGG | RT |
| RELAint8 F | RELA | 10 | CAACAGAGGCCTCCAAAAGCTG | gDNA |
| RELAex10 R | RELA | 10 | AATCCTTACCTGGCTTGGGGAC | gDNA |
| RELAex7 F | RELA | 10 | AACACTGCCGAGCTCAAGATCT | RT |
| RELAex11 R | RELA | 10 | TGCTCAGGGATGACGTAAAGGG | RT |
| SCARB1int4 F | SCARB1 | 5 | ggaggaaagccagaCTCTCCTG | gDNA |
| SCARB1int5 R | SCARB1 | 5 | AGAGTGTTCATCCTCCCAGCAC | gDNA |
| SCARB1ex4 F | SCARB1 | 5 | ACTGTGGGTGAGATCATGTGGG | RT |
| SCARB1ex7 R | SCARB1 | 5 | TTCGTTGGGTGGGTAGATGGAC | RT |
| BRCA2ex10off1F | BRCA2 OT | exon 10 OT1 | GCTGCCTACTCTGCCTACTCAG | gDNA |
| BRCA2ex10off1R | BRCA2 OT | exon 10 OT1 | gtccataggcctcaccagactg | gDNA |
| BRCA2ex10off2F | BRCA2 OT | exon 10 OT2 | TTCTGAAGGAAGACGCCTGGAG | gDNA |
| BRCA2ex10off2R | BRCA2 OT | exon 10 OT2 | gctgcaataaacatgggtgtgc | gDNA |
| BRCA2ex10off3F | BRCA2 OT | exon 10 OT3 | AATTCCCTGGCATTTAGGTTGAGC | gDNA |
| BRCA2ex10off3R | BRCA2 OT | exon 10 OT3 | GCAGAATGCAGACTTTCCCTTTCA | gDNA |
| BRCA2ex10off4F | BRCA2 OT | exon 10 OT4 | ggtgcctatcCCTGCCTTGTAT | gDNA |
| BRCA2ex10off4R | BRCA2 OT | exon 10 OT4 | TTTTACTTCGCCTTGGCACACC | gDNA |
| BRCA2ex17 Sp off1F | BRCA2 OT | exon 17 Sp OT1 | cacgcccctgtaatcccaacta | gDNA |
| BRCA2ex17 Sp off1R | BRCA2 OT | exon 17 Sp OT1 | GTGGTCTTCTTCCCACCTCCTC | gDNA |
| BRCA2ex17 Sp off2F | BRCA2 OT | exon 17 Sp OT2 | gaaatcacgccactgcattcca | gDNA |
| BRCA2ex17 Sp off2R | BRCA2 OT | exon 17 Sp OT2 | gattcacccacttttcccagcg | gDNA |
| BRCA2ex17 Sp off3F | BRCA2 OT | exon 17 Sp OT3 and KKH OT4 | tgcaaattcacagcaaagcagga | gDNA |
| BRCA2ex17 Sp off3R | BRCA2 OT | exon 17 Sp OT3 | ACATTGACCCCAGTTGCTCTCT | gDNA |
| BRCA2ex17 Sp off4F | BRCA2 OT | exon 17 Sp OT4 | TGCTGCTACTCTTTTCTGGACACT | gDNA |
| BRCA2ex17 Sp off4R | BRCA2 OT | exon 17 Sp OT4 | GGAGCAATTCCACTGATGCATCTC | gDNA |
| BRCA2ex17 KKH off1F | BRCA2 OT | exon 17 KKH OT1 | gcactcccactgccTGTATTGA | gDNA |
| BRCA2ex17 KKH off1R | BRCA2 OT | exon 17 KKH OT1 | GAGTAGGGGAAAAGAGGGGAGC | gDNA |
| BRCA2ex17 KKH off2F | BRCA2 OT | exon 17 KKH OT2 | TCAGAGACTCCATGATGCCATGTt | gDNA |
| BRCA2ex17 KKH off2R | BRCA2 OT | exon 17 KKH OT2 | gctatgttgtcctggctagagtgt | gDNA |
| BRCA2ex17 KKH off3F | BRCA2 OT | exon 17 KKH OT3 | GGGTGGTAGACAAGAAGCCTCA | gDNA |
| BRCA2ex17 KKH off3R | BRCA2 OT | exon 17 KKH OT3 | GGCACAGACAGACCACAAAAGG | gDNA |

| | | | | |
|---|---|---|---|---|
| BRCA2ex26off1F | BRCA2 OT | exon 26 OT1 | AAGCCACGTTAGCATTTTCCCTTC | gDNA |
| BRCA2ex26off1R | BRCA2 OT | exon 26 OT1 | aggcacttaatcttagagatgggct | gDNA |
| BRCA2ex26off2F | BRCA2 OT | exon 26 OT2 | tcagagatatgtcccctgccct | gDNA |
| BRCA2ex26off2R | BRCA2 OT | exon 26 OT2 | tgctttgaggatgcctttgctg | gDNA |
| BRCA2ex26off3F | BRCA2 OT | exon 26 OT3 | TTCTGCATCAGAGCTGTAAGAGGT | gDNA |
| BRCA2ex26off3R | BRCA2 OT | exon 26 OT3 | CACCAGTAGCTACAAAAAGCAGGA | gDNA |
| BRCA2ex26off4F | BRCA2 OT | exon 26 OT4 | CAGTGGGTGGTATGGGTCCTTT | gDNA |
| BRCA2ex26off4R | BRCA2 OT | exon 26 OT4 | GGTGAATGGGGTTGCAAGGATG | gDNA |
| CCNB1ex5off1F | CCNB1 OT | exon 5 OT1 | TGGGGACGGGGTTAGAAATCAC | gDNA |
| CCNB1ex5off1R | CCNB1 OT | exon 5 OT1 | TAAGCAAACAGGGAGCTGAGCT | gDNA |
| CCNB1ex5off2F | CCNB1 OT | exon 5 OT2 | CAGAACAGACGCTGGTAACACAATT | gDNA |
| CCNB1ex5off2R | CCNB1 OT | exon 5 OT2 | GCAGATAATTTTAATGCTCAGCCGC | gDNA |
| CCNB1ex5off3F | CCNB1 OT | exon 5 OT3 | GAGTCAAAGCCAATCGTCGCAA | gDNA |
| CCNB1ex5off3R | CCNB1 OT | exon 5 OT3 | TGTGGTTACTGTAGGCAAGGCA | gDNA |
| CCNB1ex5off4F | CCNB1 OT | exon 5 OT4 | TGCCATTCCCTAAACAACAGTTG | gDNA |
| CCNB1ex5off4R | CCNB1 OT | exon 5 OT4 | ggaggtgctgttaggaacccat | gDNA |
| CCNB1ex6off1F | CCNB1 OT | exon 6 OT1 | acccgcctgtaatcccagttac | gDNA |
| CCNB1ex6off1R | CCNB1 OT | exon 6 OT1 | CATTTGAGTTTTGCATGCGCGT | gDNA |
| CCNB1ex6off2F | CCNB1 OT | exon 6 OT2 | ACTGGCCTAGATGTACGTGTCT | gDNA |
| CCNB1ex6off2R | CCNB1 OT | exon 6 OT2 | atgctctactgCCTTGCTGTCA | gDNA |
| CCNB1ex6off3F | CCNB1 OT | exon 6 OT3 | ACAAGAAAGCTGTACTGGCCCT | gDNA |
| CCNB1ex6off3R | CCNB1 OT | exon 6 OT3 | TTTGTGCAAGGATGAGAGGGGA | gDNA |
| CCNB1ex6off4F | CCNB1 OT | exon 6 OT4 | cttcactgctggagggaatgga | gDNA |
| CCNB1ex6off4R | CCNB1 OT | exon 6 OT4 | tggcctccgggtttattcatgt | gDNA |
| EGFRex23off1F | EGFR OT | exon 23 OT1 | tttgatcacgccactgcattcc | gDNA |
| EGFRex23off1R | EGFR OT | exon 23 OT1 | ttagctggatatggtggtgggc | gDNA |
| EGFRex23off2F | EGFR OT | exon 23 OT2 | AGGAGGATGCTGGAGTGAGAGA | gDNA |
| EGFRex23off2R | EGFR OT | exon 23 OT2 | Aaggcccctgaatctgcattct | gDNA |
| EGFRex23off3F | EGFR OT | exon 23 OT3 | ACTTTAGTCTGCGCCAGAGGAG | gDNA |
| EGFRex23off3R | EGFR OT | exon 23 OT3 | CGGCGTCAGGTAAAACAGGTTC | gDNA |
| EGFRex23off4F | EGFR OT | exon 23 OT4 | ccttgggcccttctGTAATCCA | gDNA |
| EGFRex23off4R | EGFR OT | exon 23 OT4 | CAACCCAGATGGCTCCACTACA | gDNA |
| IL1RAPex10 Sp off1F | IL1RAP OT | exon 10 Sp and VQR OT1 | ccagtggagcctctgaagagag | gDNA |
| IL1RAPex10 Sp off1R | IL1RAP OT | exon 10 Sp and | tcagtagttcaagaccagcccg | gDNA |
| IL1RAPex10 Sp off2F | IL1RAP OT | exon 10 Sp OT2 | ATCTGGGTTGCCACAGAAGTCT | gDNA |
| IL1RAPex10 Sp off2R | IL1RAP OT | exon 10 Sp OT2 | TGGGCTGGTTAGGTAGAGGAGT | gDNA |
| IL1RAPex10 Sp off3,4F | IL1RAP OT | exon 10 Sp and VQR OT3 and 4 | TCAACTCGAGTCCAATTCCCCC | gDNA |
| IL1RAPex10 Sp off3,4R | IL1RAP OT | exon 10 Sp and | AGAAGGGCTTTTCAGGAGAGGG | gDNA |
| IL1RAPex10 VQR off2F | IL1RAP OT | exon 10 VQR OT2 | tttagtagagacggggtttcaccg | gDNA |
| IL1RAPex10 VQR off2R | IL1RAP OT | exon 10 VQR OT2 | TGATGGGGGCACTGAAGTCAAT | gDNA |
| JAG1ex9off1F | JAG1 OT | exon 9 OT1 | TGACTAGAAGGGTGGCAATGCA | gDNA |
| JAG1ex9off1R | JAG1 OT | exon 9 OT1 | CGGCCTTTTACGTTTAAGCCGT | gDNA |
| JAG1ex9off2F | JAG1 OT | exon 9 OT2 | CTCTTCCTCCCCAGCTTGTCTC | gDNA |
| JAG1ex9off2R | JAG1 OT | exon 9 OT2 | AGTACAGAAAGCGGGCCTTAGG | gDNA |
| JAG1ex9off3F | JAG1 OT | exon 9 OT3 | aggagggtggatcatctgaggt | gDNA |
| JAG1ex9off3R | JAG1 OT | exon 9 OT3 | TTAAGCCCTGTGAGCCACCTTT | gDNA |

| | | | | |
|---|---|---|---|---|
| JAG1ex9off4F | JAG1 OT | exon 9 OT4 | TGTATGTGAATGAGCGGGTGGT | gDNA |
| JAG1ex9off4R | JAG1 OT | exon 9 OT4 | AGCATGGCTTGATTCCCTGACT | gDNA |
| JAG1ex12off1F | JAG1 OT | exon 12 OT1 | AGTACTGCAGtctggcccaaat | gDNA |
| JAG1ex12off1R | JAG1 OT | exon 12 OT1 | AAGTCAAGCTGTGCTCAGGGAT | gDNA |
| JAG1ex12off2F | JAG1 OT | exon 12 OT2 | aggagaaaattcttgggcagca | gDNA |
| JAG1ex12off2R | JAG1 OT | exon 12 OT2 | cctgactctcctgaagacctgc | gDNA |
| JAG1ex12off3F | JAG1 OT | exon 12 OT3 | TGTGTAGCTTGCAAAAGACAGCA | gDNA |
| JAG1ex12off3R | JAG1 OT | exon 12 OT3 | CCCAATTTCCCAATGGCTGCTT | gDNA |
| JAG1ex12off4F | JAG1 OT | exon 12 OT4 | ttcgagcaattctcctgcctca | gDNA |
| JAG1ex12off4R | JAG1 OT | exon 12 OT4 | GTTCCTGCTTTCCCGTCACTTG | gDNA |
| LMNAex11off1F | LMNA OT | exon 11 OT1 | tggatccagcagctcaatgaca | gDNA |
| LMNAex11off1R | LMNA OT | exon 11 OT1 | ATACCGGCTGTGTGCTTAGTGT | gDNA |
| LMNAex11off2F | LMNA OT | exon 11 OT2 | GACCCTGTTGTATTGCCCCTCT | gDNA |
| LMNAex11off2R | LMNA OT | exon 11 OT2 | CGTGACAGTCTCAGGGACCAAT | gDNA |
| LMNAex11off3F | LMNA OT | exon 11 OT3 | TAAGGCACTGTGCTGAGAGCTC | gDNA |
| LMNAex11off3R | LMNA OT | exon 11 OT3 | CAGAACAAAGCAGCTGATGGCA | gDNA |
| LMNAex11off4F | LMNA OT | exon 11 OT4 | gtcccttgcctaaCACCTCAGT | gDNA |
| LMNAex11off4R | LMNA OT | exon 11 OT4 | GCCTTGGAACAGAGGATGGGAT | gDNA |
| PI4KAex9off1F | PI4KA OT | exon 9 OT1 | gaagttcaagaccagcatggcc | gDNA |
| PI4KAex9off1R | PI4KA OT | exon 9 OT1 | AGGGCGAGGTTTGCTACTGAAT | gDNA |
| PI4KAex9off2F | PI4KA OT | exon 9 OT2 | GAAACACCATGGAACGTGCACT | gDNA |
| PI4KAex9off2R | PI4KA OT | exon 9 OT2 | TATACGACCACAGGTTCTGGCC | gDNA |
| PI4KAex9off3F | PI4KA OT | exon 9 OT3 | CAGGCCTTCTTGACTGGAGGAA | gDNA |
| PI4KAex9off3R | PI4KA OT | exon 9 OT3 | GTGAGGGGAATGGAGCAGTAGT | gDNA |
| PI4KAex9off4F | PI4KA OT | exon 9 OT4 | cagaggttgcggtaagtggaga | gDNA |
| PI4KAex9off4R | PI4KA OT | exon 9 OT4 | ATCCTCTGTGTGCTCCAAGGTC | gDNA |
| PIK3CAex5off1F | PIK3CA OT | exon 5 OT1 | AGGGCTAGTTGTCTGAGGACTT | gDNA |
| PIK3CAex5off1R | PIK3CA OT | exon 5 OT1 | TATGAGTGGTCACTGGGCAGAG | gDNA |
| PIK3CAex5off2F | PIK3CA OT | exon 5 OT2 | ttgcgccaggtaagatttccag | gDNA |
| PIK3CAex5off2R | PIK3CA OT | exon 5 OT2 | tgcgggtaggggaaaatgttct | gDNA |
| PIK3CAex5off3F | PIK3CA OT | exon 5 OT3 | CATGCCCTGTCTCCAGCTCTTA | gDNA |
| PIK3CAex5off3R | PIK3CA OT | exon 5 OT3 | cctcaaaccatcctcccacctt | gDNA |
| PIK3CAex5off4F | PIK3CA OT | exon 5 OT4 | ACGTGTATCCATGTCTGTTAGCCT | gDNA |
| PIK3CAex5off4R | PIK3CA OT | exon 5 OT4 | GGTTGATCTCATGTTGCCTTGCTT | gDNA |
| RELAex6off1F | RELA OT | exon 6 OT1 | catagcccaggaacacaggtca | gDNA |
| RELAex6off1R | RELA OT | exon 6 OT1 | tgcagctgaaggtaagagaggt | gDNA |
| RELAex6off2F | RELA OT | exon 6 OT2 | AACTCAGGCTCTCAGCTTCAGG | gDNA |
| RELAex6off2R | RELA OT | exon 6 OT2 | gtgctatggtttcctggtgcac | gDNA |
| RELAex6off3F | RELA OT | exon 6 OT3 | GGCCTGACCCTTTGCTTTCATC | gDNA |
| RELAex6off3R | RELA OT | exon 6 OT3 | GTCTGCTCTGGTTTTGGCTTCC | gDNA |
| RELAex6off4F | RELA OT | exon 6 OT4 | AAGTATATTGAGCGGCCCCTCC | gDNA |
| RELAex6off4R | RELA OT | exon 6 OT4 | CTGTTGGATGCAAGGACAGCTG | gDNA |
| RELAex7off1F | RELA OT | exon 7 OT1 | tgagtgaaCAAAGTGCGGATTCTG | gDNA |
| RELAex7off1R | RELA OT | exon 7 OT1 | tgacagctgccactcattatctgt | gDNA |
| RELAex7off2F | RELA OT | exon 7 OT2 | GGCACCACAGTACAAATCAGGTG | gDNA |
| RELAex7off2R | RELA OT | exon 7 OT2 | CTTGCTCATGAAAGGCTCTGAGC | gDNA |
| RELAex7off3F | RELA OT | exon 7 OT3 | TGTAATCTCCACCCCTTCTGCAG | gDNA |
| RELAex7off3R | RELA OT | exon 7 OT3 | TTCACCACCTCATTGCACACATG | gDNA |

| | | | | |
|---|---|---|---|---|
| RELAex7off4F | RELA OT | exon 7 OT4 | CCATCTGTGACAGAGCCTTGGA | gDNA |
| RELAex7off4R | RELA OT | exon 7 OT4 | CTGGGAGGGGTGGAGCTTTAAA | gDNA |
| RELAex10off1F | RELA OT | exon 10 OT1 | CCACTTCTCTACCCACTCAGCC | gDNA |
| RELAex10off1R | RELA OT | exon 10 OT1 | atggtggcttggatcttggtga | gDNA |
| RELAex10off2F | RELA OT | exon 10 OT2 | tgttctcacagagtggagagcg | gDNA |
| RELAex10off2R | RELA OT | exon 10 OT2 | CCGAGAAATGCAGACCCAGGTA | gDNA |
| RELAex10off3F | RELA OT | exon 10 OT3 | ctgcggtctctctgtcttcaca | gDNA |
| RELAex10off3R | RELA OT | exon 10 OT3 | CCTGCGTGAATTCATAGACGCC | gDNA |
| RELAex10off4F | RELA OT | exon 10 OT4 | agacaggttctcgctctgtcac | gDNA |
| RELAex10off4R | RELA OT | exon 10 OT4 | AATTGCAAGCCGTCAGTGAAGG | gDNA |
| SCARB1ex5off1F | SCARB1 OT | exon 5 OT1 | atctggtgtgaatggggaaggg | gDNA |
| SCARB1ex5off1R | SCARB1 OT | exon 5 OT1 | ATACCCACACCTGACCCACAtg | gDNA |
| SCARB1ex5off2F | SCARB1 OT | exon 5 OT2 | GACACCATCCTCAACGCCATTG | gDNA |
| SCARB1ex5off2R | SCARB1 OT | exon 5 OT2 | CAGCCACCAAAGTATCGGGAGA | gDNA |
| SCARB1ex5off3F | SCARB1 OT | exon 5 OT3 | ACCTGCAGCTACCGAGAAACTT | gDNA |
| SCARB1ex5off3R | SCARB1 OT | exon 5 OT3 | tctcaaacagacagcgggcata | gDNA |
| SCARB1ex5off4F | SCARB1 OT | exon 5 OT4 | aatcatcccccattccccatcc | gDNA |
| SCARB1ex5off4R | SCARB1 OT | exon 5 OT4 | aactcccattccctccttctgc | gDNA |

**Table S2.** Nucleotide sequences of primers used for all PCRs.
