# Supplementary material for: CRISPR-SKIP: programmable gene splicing with single base editors
Source: Genome Biol. 2018 Aug 15;19:107. doi: 10.1186/s13059-018-1482-5 (PMC6092781; doi:10.1186/s13059-018-1482-5)
Supplement: Supplementary file 2 — Figures S1–S7. Supplemental figures. (PDF 187 kb) [file 13059_2018_1482_MOESM2_ESM.pdf]

RELA EXON 7		INTRON 6																					EXON 7					
		PAM																		SPLICE ACCEPTOR								
		C	C	C	T	T	G	T	T	T	T	C	A	C	T	G	C	C	T	C	A	G	G	T	G	C	C	C
WT	A	0.03	0.04	0.16	0.03	0.01	0.10	0.02	0.06	0.03	0.09	0.05	99.41	0.02	0.05	0.14	0.03	0.11	0.08	0.02	99.62	0.11	0.12	0.03	0.19	0.05	0.16	0.10
	C	99.84	99.87	99.77	0.27	0.22	0.01	0.32	0.36	0.45	0.29	99.88	0.10	99.88	0.30	0.02	99.85	99.79	0.41	99.88	0.06	0.03	0.04	0.31	0.05	99.79	99.65	99.67
	G	0.02	0.01	0.01	0.05	0.03	99.77	0.09	0.04	0.02	0.08	0.01	0.35	0.01	0.06	99.73	0.01	0.01	0.04	0.02	0.28	99.73	99.82	0.06	99.71	0.02	0.05	0.07
	T	0.11	0.08	0.06	99.65	99.75	0.12	99.56	99.54	99.51	99.53	0.06	0.14	0.09	99.60	0.12	0.11	0.09	99.47	0.09	0.04	0.12	0.01	99.60	0.06	0.14	0.14	0.16
SP-BE3	A	0.02	0.09	0.25	0.04	0.04	0.20	0.02	0.06	0.04	0.16	0.06	99.13	0.02	0.05	0.39	0.05	0.13	0.11	0.03	99.59	**6.26**	0.65	0.06	0.18	0.05	0.35	0.22
	C	99.81	99.77	99.66	0.28	0.29	0.03	0.33	0.36	0.38	0.37	99.84	0.16	99.87	0.32	0.03	99.83	99.75	0.35	99.87	0.07	0.50	0.07	0.26	0.06	99.71	99.41	99.48
	G	0.04	0.03	0.03	0.08	0.04	99.58	0.10	0.07	0.06	0.14	0.03	0.49	0.02	0.11	99.43	0.02	0.02	0.08	0.02	0.30	**93.00**	99.23	0.06	99.70	0.05	0.10	0.11
	T	0.13	0.11	0.07	99.60	99.64	0.18	99.54	99.52	99.52	99.33	0.07	0.22	0.09	99.53	0.16	0.11	0.10	99.46	0.08	0.04	0.24	0.05	99.62	0.06	0.20	0.14	0.18
				3'	A	A	C	A	A	A	A	G	T	G	A	C	G	G	A	G	T	C	C	A	5'			

PIK3CA EXON 5		INTRON 4																					EXON 5							
		PAM																	SPLICE ACCEPTOR											
		C	C	C	C	C	T	T	A	A	T	C	T	C	T	T	A	C	A	G	T	A	T	A	T	A	A	G
WT	A	0.16	0.15	0.20	0.20	0.09	0.07	0.09	99.86	99.70	0.18	0.03	0.13	0.09	0.15	0.01	99.34	0.12	99.30	0.24	0.02	99.64	0.04	99.68	0.05	99.70	99.51	0.03
	C	99.53	99.66	99.57	99.64	99.83	0.30	0.19	0.03	0.03	0.42	99.92	0.35	99.88	0.36	0.18	0.08	99.77	0.07	0.05	0.19	0.06	0.18	0.04	0.20	0.07	0.09	0.01
	G	0.12	0.05	0.08	0.06	0.02	0.21	0.06	0.09	0.25	0.24	0.00	0.10	0.00	0.00	0.07	0.27	0.02	0.47	99.56	0.04	0.21	0.12	0.20	0.08	0.16	0.33	99.80
	T	0.19	0.15	0.14	0.11	0.07	99.42	99.66	0.02	0.02	99.16	0.05	99.41	0.03	99.39	99.75	0.30	0.10	0.16	0.16	99.74	0.09	99.66	0.08	99.67	0.06	0.07	0.16
SP-BE3	A	0.12	0.07	0.31	0.43	0.06	0.08	0.04	99.45	99.70	0.10	0.06	0.06	0.04	0.11	0.01	99.39	0.08	99.39	**26.38**	0.03	99.61	0.02	99.68	0.12	99.65	99.49	**10.34**
	C	99.70	99.73	99.50	99.43	99.53	0.27	0.20	0.02	0.02	0.33	99.47	0.36	99.74	0.41	0.12	0.06	99.66	0.05	14.66	0.22	0.04	0.21	0.05	0.16	0.05	0.03	0.99
	G	0.05	0.06	0.05	0.05	0.01	0.15	0.05	0.48	0.25	0.19	0.42	0.05	0.08	0.08	0.10	0.36	0.13	0.39	**56.38**	0.16	0.25	0.08	0.21	0.04	0.18	0.27	**87.92**
	T	0.12	0.13	0.14	0.09	0.40	99.49	99.71	0.06	0.03	99.37	0.05	99.52	0.15	99.41	99.76	0.19	0.12	0.16	2.58	99.59	0.11	99.69	0.06	99.67	0.12	0.22	0.75
				3'	G	G	A	A	T	T	A	G	A	G	A	A	T	G	T	C	A	T	A	T	5'		

Figure S1. Expanded view of NGS analysis shown in **Fig. 2D**. Orange box: PAM sequence. Light blue: Reference sequence shown in sense orientation for clarity. Gray box: sgRNA target sequence. Deep sequencing performed on biological duplicates and averaged.

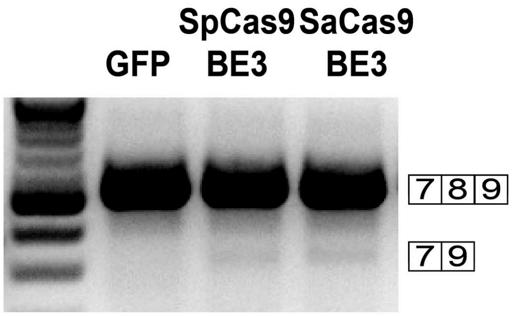

***Rela* exon 8**

Figure S2. Neuro2A cells were transfected with either SpCas9 or SaCas9-KKH C>T base editors targeting the splice acceptor of *Rela* exon 8. Analysis of exon skipping by RT-PCR demonstrated that both base editors effectively induced splicing.

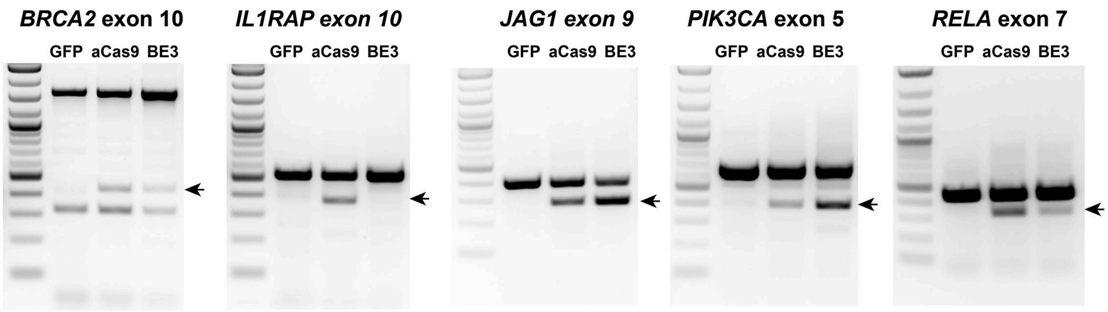

Figure S3. Comparison of CRISPR-SKIP and active SpCas9 using the same sgRNAs targeting the splice acceptor of *BRCA2* exon 10, *IL1RAP* exon 10, *JAG1* exon 9, *PIK3CA* exon 5 and *RELA* exon 7.

BRCA2 EXON 26		INTRON 25																	EXON 26									
		PAM														SPLICE ACCEPTOR												
		T	C	C	A	C	T	T	A	T	T	T	T	C	T	T	A	G	A	A	T	A	T	T	G	A	C	A
WT	A	0.21	0.21	0.20	98.35	0.30	0.08	0.02	99.70	0.09	0.03	0.14	0.17	0.14	0.13	0.11	99.68	0.24	99.56	99.48	0.14	99.71	0.11	0.13	0.32	99.31	0.11	99.45
	C	0.36	99.61	99.64	0.48	99.60	0.26	0.22	0.10	0.21	0.32	0.37	0.33	99.78	0.22	0.14	0.03	0.02	0.03	0.07	0.16	0.05	0.23	0.40	0.03	0.12	99.71	0.08
	G	0.17	0.07	0.05	0.51	0.01	0.08	0.14	0.14	0.16	0.05	0.28	0.32	0.03	0.18	0.10	0.26	99.65	0.33	0.40	0.16	0.20	0.31	0.27	99.45	0.47	0.06	0.38
	T	99.26	0.11	0.12	0.65	0.09	99.57	99.62	0.05	99.54	99.60	99.21	99.18	0.05	99.47	99.66	0.03	0.09	0.07	0.05	99.54	0.03	99.34	99.20	0.21	0.09	0.11	0.10
SP-BE3 VQR	A	0.10	0.10	0.13	99.11	0.16	0.05	0.02	99.83	0.04	0.02	0.06	0.08	0.07	0.08	0.05	99.77	**0.93**	99.63	99.63	0.05	99.80	0.06	0.07	0.23	99.41	0.07	99.61
	C	0.37	99.81	99.77	0.24	99.77	0.29	0.17	0.04	0.19	0.31	0.33	0.32	99.88	0.22	0.12	0.01	0.18	0.03	0.02	0.16	0.01	0.27	0.34	0.02	0.08	99.84	0.03
	G	0.07	0.03	0.02	0.37	0.01	0.05	0.06	0.09	0.09	0.03	0.14	0.13	0.02	0.11	0.06	0.18	**98.86**	0.31	0.32	0.06	0.16	0.13	0.13	99.67	0.44	0.02	0.32
	T	99.46	0.06	0.08	0.28	0.06	99.61	99.75	0.04	99.68	99.63	99.47	99.48	0.04	99.59	99.77	0.04	0.03	0.04	0.03	99.73	0.03	99.54	99.46	0.09	0.07	0.08	0.03
				3'	T	G	A	A	T	A	A	A	A	G	A	A	T	C	T	T	A	T	A	A	5'			

RELA EXON 10		INTRON 9																				EXON 10						
		PAM																		SPLICE ACCEPTOR								
		A	C	C	T	A	G	C	C	C	T	T	G	C	C	C	T	A	C	A	G	G	A	C	C	C	A	C
WT	A	98.57	0.10	0.09	0.10	99.43	0.20	0.09	0.05	0.04	0.04	0.03	0.16	0.23	0.16	0.12	0.05	98.42	0.06	99.26	0.23	0.15	98.49	0.06	0.10	0.05	97.38	0.11
	C	0.54	99.79	99.75	0.30	0.16	0.11	99.72	99.71	99.77	0.31	0.35	0.07	99.06	99.68	99.71	0.24	0.83	99.83	0.12	0.07	0.03	0.63	99.84	99.73	99.86	1.44	99.75
	G	0.69	0.03	0.04	0.13	0.26	99.61	0.04	0.07	0.07	0.17	0.18	99.67	0.13	0.08	0.01	0.11	0.45	0.01	0.54	99.58	99.72	0.66	0.01	0.05	0.01	1.08	0.03
	T	0.20	0.09	0.12	99.47	0.15	0.08	0.14	0.18	0.12	99.48	99.44	0.11	0.57	0.09	0.16	99.60	0.31	0.10	0.08	0.12	0.10	0.23	0.09	0.12	0.08	0.10	0.11
SA-BE3 KKH	A	97.89	0.20	0.18	0.17	99.06	0.37	0.19	0.07	0.14	0.03	0.07	0.46	0.42	0.40	0.19	0.12	97.64	0.20	99.07	**46.61**	**48.95**	96.83	0.08	0.22	0.12	95.64	0.14
	C	0.79	99.64	99.57	0.33	0.32	0.20	99.54	99.46	99.51	0.29	0.37	0.14	98.68	99.29	99.52	0.23	1.30	99.67	0.23	0.68	2.30	1.59	99.79	99.54	99.76	2.51	99.66
	G	0.95	0.07	0.09	0.27	0.37	99.25	0.05	0.16	0.15	0.21	0.29	99.18	0.16	0.22	0.02	0.26	0.60	0.03	0.58	**51.80**	**47.52**	1.01	0.03	0.08	0.02	1.61	0.08
	T	0.37	0.09	0.16	99.23	0.25	0.18	0.23	0.31	0.20	99.47	99.27	0.23	0.73	0.09	0.27	99.39	0.46	0.10	0.11	0.91	1.23	0.56	0.10	0.15	0.10	0.23	0.12
							3'	G	G	G	A	A	C	G	G	G	A	T	G	T	C	C	T	G	G	G	T	5'

Figure S4. Expanded view of NGS analysis shown in **Fig. 5C and 5D**. Orange box: PAM sequence. Light blue: Reference sequence shown in sense orientation for clarity. Gray box: sgRNA target sequence. Deep sequencing performed on biological duplicates and averaged.

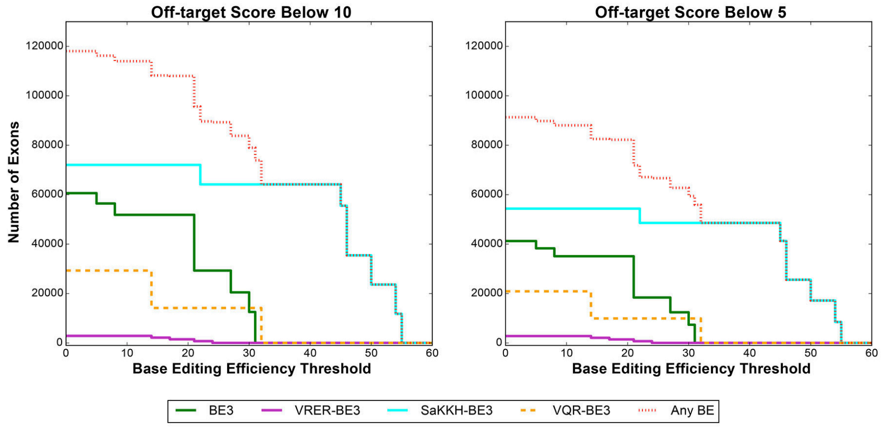

Figure S5. Estimation of the number of exons that can be targeted by each base editor with subplots filtered by the maximum allowed off-target score. The *y*-axis denotes the number of exons that can be targeted with estimated efficiency of modifying intronic flanking G at or above the corresponding value on the *x*-axis.

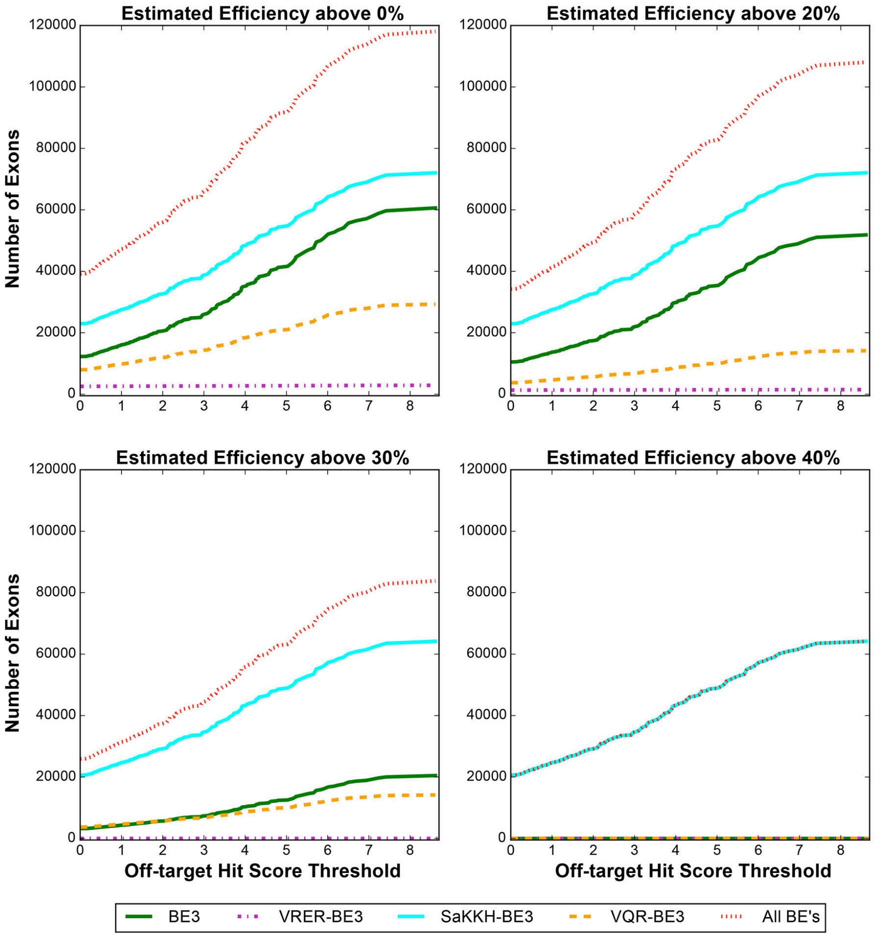

Figure S6. Estimation of the number of exons that can be targeted by each base editor with subplots filtered by estimated efficiency of editing the flanking G nucleotide. The *y*-axis denotes the number of exons that can be targeted with maximum off-target score at or below the corresponding value on the *x*-axis.

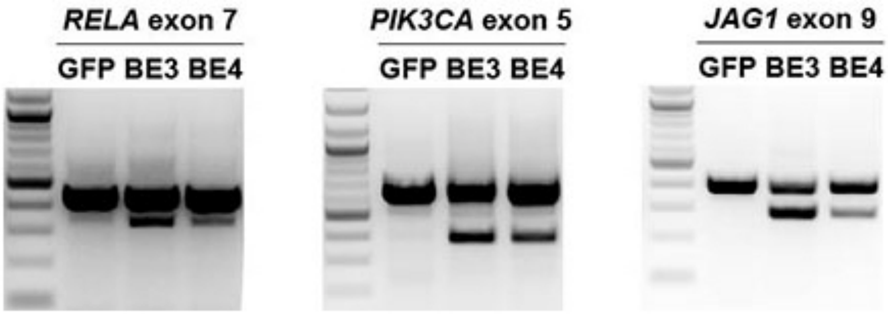

Figure S7. Comparison of CRISPR-SKIP using the C>T base editors BE3 or BE4 for inducing skipping of *PIK3CA* exon 5, *RELA* exon 7, and *JAG1* exon 9 by RT-PCR analysis.
